# Heat up, silence on: *IDO1* gene silencing in THP-1-derived dendritic cells triggered by magnetic hyperthermia

**DOI:** 10.1007/s00262-025-04148-3

**Published:** 2025-08-23

**Authors:** Daniela Ferreira, Laura Asín, Javier Idiago-López, Valeria Grazú, Jesús M. de la Fuente, Raluca M. Fratila, Pedro V. Baptista, Alexandra R. Fernandes

**Affiliations:** 1 Associate Laboratory i4HB - Institute for Health and Bioeconomy, NOVA School of Science and Technology, NOVA University Lisbon, 2819-516 Caparica, Portugal; 2 UCIBIO – Applied Molecular Biosciences Unit, Department of Life Sciences, NOVA School of Science and Technology, NOVA University Lisbon, 2819-516 Caparica, Portugal; 3 Instituto de Nanociencia y Materiales de Aragón, INMA (CSIC-Universidad de Zaragoza, C/Pedro Cerbuna 12, 50009 Saragoza, Spain; 4 Centro de Investigación Biomédica en Red de Bioingeniería, Biomateriales y Nanomedicina (CIBER-BBN), Saragoza, Spain; 5 Department of BioChemistry and Molecular and Cellular Biology/Institute for Biocomputation and Physics of Complex Systems, University of Zaragoza, Saragoza, Spain; 6 Departamento de Química Orgánica, Facultad de Ciencias, C/Pedro Cerbuna 12, 50009 Saragoza, Spain

**Keywords:** Dendritic cells, Indoleamine 2,3-dioxygenase 1, Gene silencing, Transfection, Magnetic hyperthermia

## Abstract

Dendritic cells (DCs) are well-known antigen-presenting cells which have an important role in cancer immunomodulation due to the effective regulation of immune responses in the tumor microenvironment (TME). Indoleamine 2,3-dioxygenase 1 gene (*IDO1*) is upregulated in many types of cancers and associated with a poor prognosis, contributing to an immunosuppressive TME. *IDO1* silencing in DCs is considered a promising new strategy in gene therapy owing to their capability to regulate T cells function and activation. This study focuses on the use of magnetic hyperthermia (MH) combined with bioorthogonal chemistry to promote siRNA transfection against *IDO1* in THP-1-derived DCs. Magnetic nanoparticles (MNPs) functionalized with cyclooctyne moieties were attached by strain-promoted azide-alkyne cycloaddition to DCs membranes engineered to express artificial azide receptors. Upon the application of an alternating magnetic field, the MNPs generate heat and trigger the thermal disruption of the cell membrane. Results show that *IDO1* gene expression decreases around 70% in THP-1-derived DCs, and that the MH-promoted transfection presents a silencing effect comparable to that attained with a gold standard Lipofectamine reagent, but with less cytotoxicity. Additionally, *IDO1* silencing promotes the upregulation of mRNA levels of pro-inflammatory cytokines *IL-6*, *TNF-α* and *IL-12A*, and the downregulation of anti-inflammatory cytokine *IL-10*, providing a more immunogenic state which may lead to THP-1-derived DCs activation for future T cells antitumor response. Our findings reveal the potential of MH-mediated transfection to enhance the intracellular delivery of silencing moieties in cells difficult to transfect, such as DCs, as well as demonstrate the possibility of silencing *IDO1* gene to overcome the immunosuppressive barrier imposed by the TME for cancer therapy.

**Supplementary Information:**

The online version contains supplementary material available at 10.1007/s00262-025-04148-3.

## Introduction

Dendritic cells (DCs) are a heterogeneous group of immune cells and specific antigen-presenting cells which play an important role in the modulation of T cell-dependent immunity in the adaptive and innate responses [1, 2]. Currently, DCs have been used in in vitro and in vivo studies, and in clinical trials as primary targets for autoimmune disorders, infectious diseases, allergies, and cancer [3]. Primary DCs are extremely difficult to manipulate in vitro and in vivo owing to the lower number of cells obtained [4]. Over the years, the identification of different maturation stages and phenotypes of DCs has been explored, but their specific characteristics and functions in a specific biological system still require a better understanding [4–7]. Hence, monocytes or other bone marrow-derived stem cells and myeloid leukemia-derived cell lines have been used as DCs precursors for isolation and differentiation in vitro to increase knowledge of the role of DCs in cancer immunomodulation [6–8]. DCs have been employed for the development of vaccine delivery systems, gene therapy, and cancer immunotherapy strategies. For instance, electroporation [9] and viral approaches [10] are the most common transfection methods for DCs, but still present limitations (e.g., low transfection efficiency and cytotoxicity) [11]. Since DCs are difficult to transfect, alternative non-viral nanoscale approaches, such as organic and inorganic nanoparticles, must be addressed to improve gene delivery [10].

Indoleamine 2,3-dioxygenase 1 (IDO1) is a cytosolic heme-containing enzyme involved in the degradation of tryptophan to kynurenine and in the modulation of innate immune responses [12–14]. Recently, IDO-expressing DCs have been described as an acquired mechanism of immune tolerance due to the ability to suppress the function of effector T and natural killer cells (NK cells) and to induce the expansion of regulatory T cells (Treg cells) and myeloid-derived suppressor cells (MDSCs), promoting neovascularization of solid tumors [1, 15, 16]. IDO1 expression has been reported in cancer cells and in the stroma surrounding the tumor microenvironment (TME) (e.g., endothelial cells, fibroblasts, immune cells, and mesenchymal cells), as well as in peripheral blood mononuclear cells of cancer patients [14, 17, 18]. In fact, IDO1 is an important immunotherapy target in cancer due to the induction of anticancer responses, and IDO1 inhibitors (e.g., indoximod) have been evaluated in clinical trials [19–21]. However, IDO1 expression patterns and the exact mechanism of function are still unclear [22–24]. Nevertheless, silencing of *IDO1* in DCs represents a powerful novel strategy in gene therapy due to their ability to regulate T cells function and activation. Immature DCs (iDCs) and mature DCs (mDCs) have an essential function in the TME, decreasing T cells activity owing to the induction of immune suppression that promotes tumor progression and survival [19, 24].

The delivery of therapeutic nucleic acids (TNAs), such as small interfering RNAs (siRNA), short hairpin RNAs, and antisense oligonucleotides, has been reported as a proper strategy to silence specific intracellular targets to overcome tumor development and metastasis [25–27]. No single transfection approach for delivering TNAs suits all cell types and experimental goals. The optimal transfection strategy is determined by many factors, such as (i) the origin of cells; (ii) the type of nucleic acids to be transfected; (iii) the transfection efficiency; (iv) the host-cells cytotoxicity; (v) the experimental budget; and (vi) the availability of required facilities [27]. Subsequently, innovative, less cytotoxic, and more effective approaches for siRNA delivery into DCs compared to commercial transfection reagents or electroporation methods are required [28, 29].

Recently, we proved that cell membrane-localized magnetic hyperthermia (MH) improves transfection of *antisense* siRNA for silencing the *GFP* gene in MCF7 breast adenocarcinoma cell line [30]. Of note, we also demonstrated that the localized heat generated by magnetic nanoparticles (MNPs) confined to the cell membrane does not have a detrimental effect on the cell viability (sub-lethal MH). This on-demand modification of cell membrane permeability allows the MH-mediated intracellular transport of biologically relevant (bio)molecules, promoting direct intracellular delivery and thus overcoming traditional endocytosis pathways and avoiding endosomal entrapment. Indeed, membrane-localized MH does not directly regulate the intracellular pathway but offers a safer and non-cytotoxic method for the direct intracellular delivery of silencing moieties [30], contrary to other strategies that rely on endosomal disruption (e.g., ultrasound [31], light [32]), which can be inefficient and cytotoxic [31]. Although the effect of MH as promoting the transfection of therapeutics moieties in cancer cells has been previously reported [30, 33, 34], it has never applied in more difficult to transfect cells, such as DCs. In this work, we show that this strategy can be applied with success in DCs derived from the THP-1 acute monocytic leukemia cell line as a model for the delivery of silencing strategies targeting the *IDO1* gene that could enhance immunomodulation for cancer therapeutics.

**Fig. 1:**
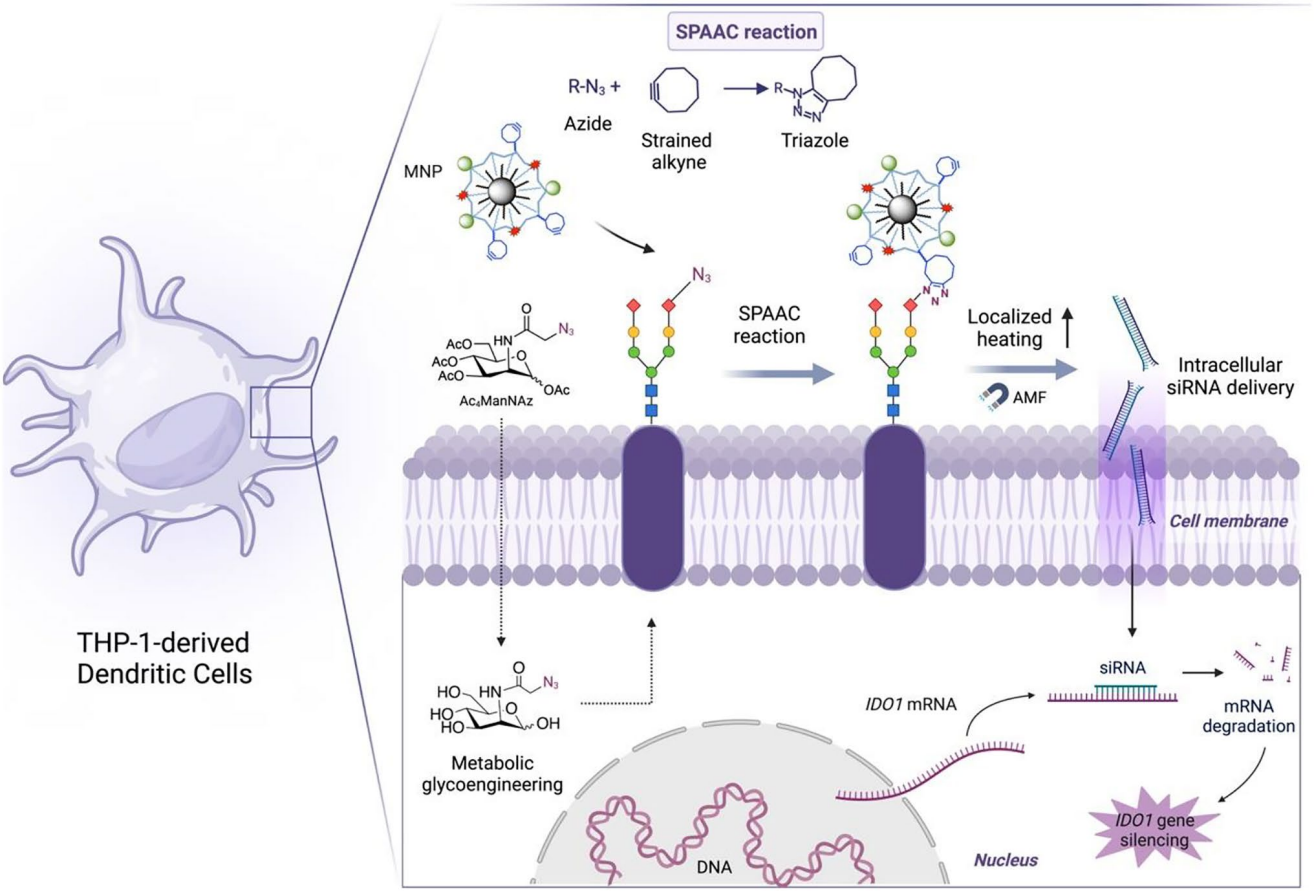
Schematic overview of the MH-mediated siRNA transfection. The magnetic nanoheaters (MNPs functionalized with cyclooctyne) were attached to the membrane of THP-1-derived DCs previously exposed to metabolic glycoengineering to express unnatural azide bioorthogonal reporters. Upon application of the AMF, the localized changes in cell membrane fluidity were used for intracellular delivery of siRNA for *IDO1* gene silencing

Our cell membrane-localized MH approach relies on the creation of hotspots on cell membranes during the application of an alternating magnetic field (AMF), due to the presence of MNPs attached to unnatural azide groups expressed on the cell membrane via strain-promoted azide-alkyne cycloaddition (SPAAC) bioorthogonal chemistry. The general concept for MH-mediated transfection using MNPs immobilized on the cell membrane is represented in Fig. 1.

## Materials and methods

### Magnetic nanoparticles (MNPs) synthesis, functionalization and characterization [35]

#### MNPs synthesis

Monodisperse spherical iron oxide MNPs with a core diameter of 13 nm were obtained in organic phase and transferred to aqueous phase to yield MNPs@PMAO according to the protocol optimized by our group [36, 37]. The concentrations of MNPs indicated represent the concentrations of iron, determined in *MNP characterization*.

#### Functionalization of the MNPs with PEG and cyclooctyne [37]

MNPs@PMAO@PEG were obtained by incubating 1 mg of MNPs@PMAO with 16.7 mg (129 μM) of $\alpha$-methoxy-ω-amino-poly(ethylene glycol) (MW = 750 Da, Rapp Polymere, Tuebingen, Germany) in 420 μL of sodium tetraborate buffer (SSB) pH 9 (50 mM of boric acid and 50 mM of sodium borate). A solution containing 6.25 mg of N-(3-dimethylaminopropyl)-N′-ethylcarbodiimide hydrochloride (EDC·HCl) dissolved in 20 μL of SSB buffer (50 mM, pH 9) was prepared 2 times, and one volume of solution was added to the previous mixture and shaken in a rotator disk during 30 min in the absence of light. Then, a second addition of EDC·HCl solution was done and the reaction mixture was incubated for another 3 h in the rotator disk. Finally, the excess of ligand was removed by washing the MNPs with Milli-Q water in a centrifugal filter with a 100 kDa molecular weight cut-off membrane (Millipore) at 8497×g for 15 min (each wash and repeated for a total of six washing steps).

MNPs@PMAO@PEG@CO were obtained by incubating 0.5 mg of MNPs@PMAO@PEG with 273 μL of cyclooctyne (5 mM) in 1.2 mL of SSB (50 mM, pH 9). Two additions of 3.125 mg of EDC dissolved in 10 μL of SSB (50 mM, pH 9) were done in the same incubation conditions previously described. Finally, the ligand excess was removed by washing the MNPs as previously described for the MNPs@PMAO@PEG. The cyclooctyne derivative used for the functionalization (11-(cyclooct-2-yn-1-yloxy)-3,6,9-trioxaundecylamine) was prepared following the procedure described in our previous work [37] (see Fig. S1).

#### MNPs characterization

*Iron concentration.* The iron concentrations of the nanoparticle suspensions were determined using a standard colorimetric method. Three replicas of samples of 5 μL and 45 μL of solvent (hexane or water) were incubated with 100 μL of aqua regia solution (HCl/HNO_3_; 3/1) at 60 °C for 15 min, after which Milli-Q water was added until a final volume of 500 μL. 50 μL of the final total volume of each solution was transferred to a 96-well plate. Then, 60 μL of a solution consisting of 50 μL of 4 N KOH and 10 μL of 0.25 M Tiron (1,2-dihidroxybenzen-3,5-disulfonic acid), and 100 μL of 0.2 M Na_3_PO_4_ (pH = 9.7) were added. The measurement of the sample absorbance at 480 nm was carried out on a Thermo Scientific Multiskan™ GO or a Biotek Synergy H1 UV/Vis microplate spectrophotometer. A similar protocol was followed using iron standard solutions (100–800 µg/mL) to obtain a calibration curve.

*Dynamic light scattering (DLS) and ζ-potential measurements.* DLS and ζ-potential measurements were performed on a Malvern Zetasizer ZS-NANO at 25 °C and pH 7. Samples were prepared at a concentration of 0.05 mg Fe/mL in Milli-Q water. Each sample was measured five times, combining 10 runs per measurement. Results were treated using the Malvern software Zetasizer Nano 7.13.

*Agarose gel electrophoresis.* For the agarose gel electrophoresis, a solution of 1% agar in 0.5× Tris–borate-EDTA (TBE) was prepared. MNPs samples mixed with 20% glycerol: 0.5× TBE were loaded in the gel, and an electric field of 90 V was applied for 45 min.

*Transmission electron microscopy (TEM).* TEM images were obtained on a Tecnai T20 microscope (FEI) operating at 200 kV (Laboratorio de Microscopias Avanzadas LMA, University of Zaragoza). A single drop (10 μL) of a solution (0.1 mg Fe/mL) of the different nanoparticles was placed onto a copper grid coated with a carbon film (Electron Microscopy Sciences). The grid was left to dry in the air for several hours at room temperature (RT). Particle size distribution was evaluated from several micrographs using Digital Micrograph software. At least 200 particles were selected for analysis, which resulted in a stable size distribution statistic.

*Heating efficiency characterization.* The specific absorption rate (SAR) was calculated using a DM100 series magnetic field applicator (nanoscale Biomagnetics, Zaragoza, Spain). Samples were measured at a concentration of 1 mg Fe/mL in Milli-Q water. The AMF was applied for 3 min using a field amplitude of 23.9 kA/m and a frequency of 418 kHz, while the temperature was recorded using an optic fiber sensor incorporated in the equipment. The SAR value was calculated according to the temperature slope obtained at the beginning of the measurement.

*Thermogravimetric analysis (TGA).* TGA was performed on lyophilized MNPs samples using a TA STD 2960 simultaneous DTA-DTGA instrument in air, at a heating rate of 10 °C/minute.

### Cell culture maintenance

THP-1 acute monocytic leukemia cell line (ATCC, USA) was grown in RPMI 1640 culture medium (Roswell Park Memorial Institute 1640), supplemented with 10% (v/v) Fetal Bovine Serum (FBS) 1% (v/v) Penicillin (100 U/mL)-1% (v/v) Streptomycin (100 mg/mL) and 1% MEM non-essential amino acids, maintained at 37 °C, under 5% CO_2_ and 99% relative humidity. Upon growth to confluency, cells were collected and stained with 0.4% Trypan Blue solution, counted using a hemocytometer, and cultured into fresh medium. All cell culture maintenance reagents were purchased from Gibco (ThermoFisher Scientific, USA). For differentiation of THP-1-derived iDCs, 2 × 10^5^ cells/mL of THP-1 were grown in culture medium supplemented with 25 ng/mL of rh IL-4 (recombinant human Interleukin 4) and rh GM-CSF (recombinant human Granulocyte Macrophage Colony-Stimulating Factor). Cells were incubated for five days at 37 °C, under standard cell culture conditions, and the culture medium of THP-1-derived iDCs was replaced every two or three days, supplemented with 25 ng/mL of IL-4 and GM-CSF. For THP-1-derived mDCs, 2 × 10^5^ cells/mL of THP-1-derived iDCs were grown in culture medium without FBS, supplemented with 25 ng/mL of rh IL-4 and rh GM-CSF, 200 ng/mL of ionomycin, and 25 ng/mL of rh TNF-α (recombinant human tumor necrosis factor α) for three days at 37 °C, 5% (v/v) C${\text{O}}_{2}$ and 99% of relative humidity. After this incubation period, the THP-1-derived mDCs culture medium was replaced with RPMI supplemented with FBS and respective growth factors [38–40]. All cell culture supplements for THP-1 differentiation in DCs were purchased from Sigma-Aldrich, USA.

### Characterization of cells’ membrane superficial receptors expression

The expression of the cells membrane receptors was measured using suitable monoclonal antibodies conjugated with phycoerythrin (PE) from Abcam, UK. In a microfuge tube, 2 × 10^5^ cells were placed with phosphate-buffered saline (PBS) supplemented with 10% FBS and the respective monoclonal antibody: Monoclonal IgG1 (Isotype Control), Monoclonal CD80 (costimulatory molecule CD24), Monoclonal CD83 (signal of maturation) and CD86 (costimulatory molecule CD24); incubated for 30 min at 4 °C, in a dark room. At last, cells were centrifuged to remove the supernatant, washed twice and resuspended in PBS. Flow cytometry was analyzed on an Attune® Acoustic Focusing Flow Cytometer and Attune® Cytometric software (Life Technologies, ThermoFisher Scientific, USA). The fluorescence of PE (λ_abs_ 488 nm/λ_em_ 575 nm) was acquired using filter BL2 (excitation and emission range wavelengths of 488 nm and 561 to 587 nm, respectively).

### Cell membrane labeling with MNPs in THP-1 cells and THP-1-derived DCs [35]

#### Expression and characterization of azide groups

Cells were seeded at a density of 1.5 × 10^5^ cells/well on a 24-well plate in supplemented RPMI and incubated with 10, 20, 50, and 100 µM of Ac_4_ManNAz (Jena Bioscience, Germany) for 48 h at 37 °C, under standard cell culture conditions. Upon incubation, cells were washed twice with DPBS (PBS with extra Ca^2+^ and Mg^2+^). Then, cells were incubated for 30 min at 37 °C in serum-free RPMI containing 20 μM DBCO-PEG_4_-5/6-Sulforhodamine B (Jena Bioscience, Germany). Cells without Ac_4_ManNAz pre-treatment were used as control. Lastly, the cells were washed twice (Gibco, ThermoFisher Scientific, USA), resuspended in PBS and analyzed by flow cytometry. The fluorescence of Sulforhodamine B (λ_abs_ 568 nm/λ_em_ 585 nm) corresponding to red fluorescence was acquired using filter BL2 (excitation and emission range wavelengths of 488 nm and 561 to 587 nm, respectively).

#### Interaction of MNPs with cells treated and non-treated with Ac_4_ManNAz

*Confocal microscopy.* Cells were seeded at a density of 1.5 × 10^5^ cells/well on 12 mm coverslips treated with 0.01% (v/v) of poly-L-lysine solution (Merck Millipore, Germany) inside a standard 24-well plate and incubated with 100 μM of Ac_4_ManNAz for 48 h at 37 °C, under 5% CO_2_ and 99% relative humidity. Following 48 h, cells were washed twice with DPBS and incubated with 20 μM of DBCO-PEG_4_-5/6-TAMRA (Jena Bioscience, Germany) as a control to ensure the correct expression of azide groups on the cell membrane, and with 100 μg_Fe_/mL of MNPs for 30 min; or 10 μg_Fe_/mL of MNPs for 10 min, in RPMI without FBS at 37 °C. Control experiments were also performed using cells without Ac_4_ManNAz pre-treatment. After this step, cells were swiftly washed twice with DPBS and fixed with 4% (v/v) of paraformaldehyde for 20 min at RT and under light protection. After fixation, cells were washed twice with PBS and stained for 15 min at RT with 7.5 μg/mL of Hoechst 33258 (Invitrogen, ThermoFisher Scientific, USA). Then, two more washing steps with PBS were performed, and the coverslips were mounted on glass microscope slides using ProLong™ Glass Antifade Mountant (ThermoFisher Scientific, USA). Confocal microscopy images were acquired using a Confocal Microscope Zeiss LSM 710 (Zeiss, Germany) with a 40× oil immersion objective. TAMRA and Hoechst fluorophores were laser-excited at 561 and 405 nm, respectively. Laser intensity and sensitivity values were optimized and maintained constant for each image capture for the samples with the same amount of fluorophore. Z-stack images were obtained with a 1024 × 1024 resolution and analyzed with the open access software platform FIJI (ImageJ).

*Flow cytometry.* Cells were seeded at a density of 1.5 × 10^5^ cells/well in 24-well plates and incubated with 100 μM of Ac_4_ManNAz for 48 h at 37 °C, under standard cell culture conditions. After 48 h, cells were then washed twice with DPBS. Samples were incubated for 10 min with 10 μg_Fe_/mL of MNPs in RPMI without FBS. Control experiments were also carried out using cells without Ac_4_ManNAz pre-treatment, and cells were incubated for 30 min at 37 °C with 20 μM of DBCO-PEG_4_-5/6-TAMRA as a positive control. Cells were washed twice with PBS and resuspended in PBS and analyzed by flow cytometry. The fluorescence of TAMRA (λ_abs_ 560 nm/λ_em_ 565 nm) corresponding to red fluorescence was acquired using filter BL2 (excitation and emission range wavelengths of 488 nm and 561 to 587 nm, respectively).

#### Magnetic hyperthermia for siRNA transfection (silencing of Indoleamine 2,3-dioxygenase) [30]

THP-1-derived DCs cells were seeded at a density of 3 × 10^5^ cells/well and treated with 100 μM of Ac_4_ManNAz before the MH experiment for 48 h at 37 °C, under 5% CO_2_ and 99% relative humidity. After 24 h, 10 ng/µl of LPS (Lipopolysaccharides from *Escherichia coli* O111:B4, Sigma-Aldrich, USA) was added to cells to induce expression of *IDO1* gene. Following 48 h with Ac_4_ManNAz treatment, cells were washed twice with PBS and incubated for 10 min with 10 μg_Fe_/mL of MNPs in RPMI without FBS. Then, cells were washed twice with PBS, centrifuged, and resuspended in fresh supplemented medium. The cells suspension was introduced into the adapted glass vial suitable for the MH equipment, and 20 nM of siRNA (Dharmacon™, Horizon Discovery, Germany) complementary to *IDO1* transcript (GenBank NM_002164.6) was added. MH was applied for 30 min with pulses (each five minutes, sixty-second pause), 300 Gauss, 418 kHz in a MH applicator (DM100 Series device; nB nanoScale Biomagnetics, Spain). As a control of gene silencing, 20 nM of siRNA was added with Lipofectamine™ RNAiMAX (Invitrogen, ThermoFisher Scientific, USA; hereafter designated as Lipofectamine for simplicity), according to manufacturer’s recommendations. After transfection with the siRNA, cells were counted and seeded on 96-well plate for cell viability assessment and on a new 24-well plate for RNA extraction at 37 °C, under standard cell culture conditions.

### siRNA transfection with Lipofectamine

THP-1-derived DCs were seeded at a density of 3 × 10^5^/well in a 24-well plate and incubated for 48 h at 37 °C, under 5% CO_2_ and 99% relative humidity. After 48 h, 20 nM of siRNA was mixed with the cationic lipid reagent Lipofectamine in RPMI (0% FBS and without antibiotics) and incubated for 5 min in a microcentrifuge tube according to the manufacturer’s recommendation. Next, siRNA–Lipofectamine complex was added to cells in RPMI (final 8% FBS) and incubated for 24 h. After incubation, cells were recovered, total RNA was extracted, and the efficiency of siRNA transfection for *IDO1* silencing was evaluated by RT-qPCR.

### Gene expression analysis

Expression of *IDO1 gene* and cytokines IL-6, IL-10, TNF-α (*IL-6, IL-10*, and *TNFA* genes, respectively), and *IL-12A* was verified via RT-qPCR assay following 24 h of gene silencing experiment. RNA was extracted from samples using the TRIsure™ reagent (Bioline, UK) according to the manufacturer’s guidelines. Total RNA extracted from THP-1-derived DCs was reverse transcribed to cDNA using the NZY M-MuLV First-Strand cDNA Synthesis kit (Nzytech, Portugal). RT-qPCR was performed using NZYSupreme qPCR Green Master Mix (2×) (Nzytech, Portugal) according to manufacturer’s protocol in a Qiagen Rotor-Gene Q cycler (Qiagen, Germany). The following conditions were used for *IDO1*: initial denaturation at 95 °C for 5 min; 35 cycles of 95 °C for 30 s, Tm 58 °C for 30 s, 72 °C for 15 s; for *IL-6*, *IL-10*, *TNFA* and *IL-12A*: initial denaturation at 95 °C for 5 min; 40 cycles of 95 °C for 30 s, Tm 53 °C for 30 s, 72 °C for 30 s. Primers forward and reverse were purchased from STABVIDA, Portugal. Gene expression was evaluated according to the 2^−ΔΔCt^ method, using the *18S* ribosomal gene as reference [41]. The primers sequences and siRNA target sequence against *IDO1* gene were provided in Supplementary Information (Table S1).

### Cell viability assay

Cell viability was evaluated by MTS assay (CellTiter 96® AQueous One Solution Cell Proliferation Assay Kit, Promega, USA) according to the manufacturer’s instructions. MTS assay was performed to determine the cytotoxicity of cells incubated with Ac_4_ManNAz and MNPs. Cells were seeded at 6 × 10^4^ cells/well in a 96-well plate and incubated with 100 μM of Ac_4_ManNAz at 37 °C, under standard cell culture conditions. After 48 h of Ac_4_ManNAz treatment, cells were washed twice with PBS and incubated for 30 min with 100 μg_Fe_/mL of MNPs in RPMI without FBS at 37 °C, under standard cell culture conditions. Cells were then washed twice with PBS, and the MTS solution (1:5 v/v in RPMI) was added to the cells and incubated for 1 h and 30 min at 37 °C, under 5% CO_2_ and 99% relative humidity. MTS assay was also used to evaluate the viability of THP-1-derived DCs following the transfection of siRNA mediated by MH. Post siRNA transfection, cells were placed at approximately 5 × 10^4^ cells/well in a 96-well plate, MTS solution was added to the cells and incubated for 2 h at 37 °C, under standard cell culture conditions. The absorbance was measured at 490 nm using Infinite M200 (Tecan, Switzerland). Cell viability was normalized to control cells (cells with culture medium).

### Statistical analysis

Data were analyzed using GraphPad Prism 8.0 (GraphPad Software, San Diego, USA). Two-way ANOVA with Tukey’s multiple comparison test and unpaired parametric t test with Welch’s correction were used to evaluate differences between groups. They were considered statistically significant at p-value < 0.05. Data are the mean value of at least three independent assays with at least two technical replicates, and the errors are calculated by the standard error of the mean.

## Results and discussion

### THP-1 acute monocytic leukemia cell line as DCs-model

The immunomodulation of DCs is required to overcome the immune suppression in cancer therapy. DCs can activate inflammatory cytokines such as TNF-α, IL-12 and interferon-γ, costimulatory molecules (e.g., CD80, CD86, CD40), necessary for cytotoxic effect, or anti-inflammatory cytokines (e.g., IL-10, IL-13), transforming growth factor β (TGF-β) and other inhibitory receptors that can mediate signaling pathways involved in cancer [7, 42]. In Table 1, the main differences between iDCs and mDCs are represented.

**Table 1:** Morphological and immunological characteristics of iDCs and mDCs [43]

Characteristics	iDCs	mDCs
Cell morphology	Round and smooth surface; change shape and migrate to lymph nodes induced by cytokines after phagocytosis	Rough surface with multiple pseudopodia; migrate to lymph nodes using active movement of dendrites at higher speed than iDCs
Cell phenotype	Lower levels of CD80, CD83, CD86 and MHC II; secrete lower levels of cytokines (IL-12, IL-10 and TNF-α); express chemokine receptors (CCR7) and are induced by chemokines (CCL19 and CCL21) after phagocytosis	High levels of costimulatory molecules (CD80, CD83, CD86); secrete higher levels of immunostimulatory cytokines (TNF-α, IL-12 and IL-10); express increase levels of fascin-1, an actin-bundling protein
Cell behavior	Ability to detect and phagocytize pathogens and antigens; initiate innate and adaptative immunity responses	Ability to produce higher levels of immunostimulatory cytokines and to behave as antigen-presenting cells for T cells activation

Some myeloid leukemia-derived cell lines (e.g., THP-1, U937, and MUTZ-3) stimulated by specific growth factors and cytokines can resemble a differentiated DCs-model [38–40, 44].

**Fig. 2:**
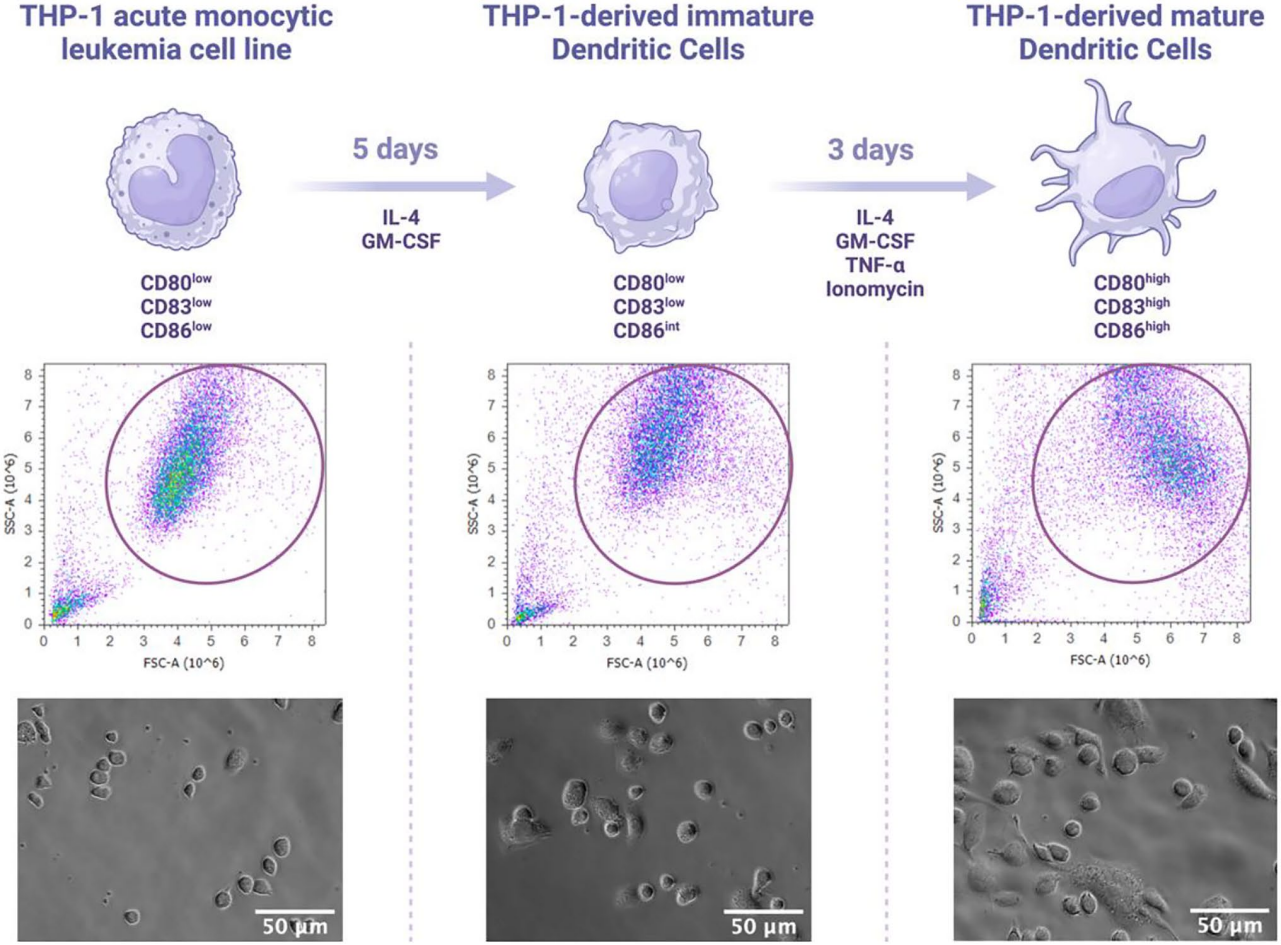
Differentiation of THP-1 cell line in a DCs-model. THP-1 cells were incubated with IL-4 and GM-CSF for five days under standard cell culture conditions to differentiate into THP-1-derived iDCs. Then, THP-1-derived iDCs were induced with IL-4, GM-CSF, TNF-α, and ionomycin for three days in serum-free media under standard cell culture conditions to mature into THP-1-derived mDCs. A shift in the flow cytometry plots’ forward scatter (size) and side scatter (complexity) was observed due to the differentiation and maturation of THP-1-derived DCs

THP-1 cells have been reported as an appropriate cell model owing to their similar immunophenotype, functional characteristics, and typical morphology comparable to iDCs and mDCs when induced with IL-4, GM-CSF, TNF-α, and ionomycin (Fig. 2) [45]. The differentiation stage and morphology of THP-1 cells are similar to those observed in human primary monocytes and macrophages. THP-1 cells have a large, round single-cell morphology, express specific monocytic markers, and have an advantage due to their easy manipulation in vitro [44].

After finishing the incubation time necessary for cell differentiation in the presence of the respective growth factors and cytokines, the expression of membrane receptors in THP-1-derived DCs was characterized by flow cytometry using monoclonal antibodies conjugated with fluorescent dye (PE). CD80 and CD86 are costimulatory molecules important for T cells activation and regulation, mostly expressed in mDCs [42, 43]. Furthermore, the presence of CD83 is a signal of DCs maturation, whose expression can be detected in activated immune cells and antigen-presenting cells [46]. The results obtained in Fig. S2 show a higher expression of CD80, CD83, and CD86 in THP-1-derived mDCs (see the histogram in Fig. S2a), proving that THP-1 cell line can be differentiated into mDCs in agreement with the literature. Compared to THP-1 cells, the expression of CD80 (Fig. S2b) increases threefold and 16-fold in THP-1-derived iDCs and in THP-1-derived mDCs, respectively. A significant increase of CD83 expression (Fig. S2c) around fivefold and 25-fold was observed in THP-1-derived iDCs and in THP-1-derived mDCs compared to THP-1 cells, which confirms DCs maturation. Moreover, in Fig. S2d a higher expression of CD86 was observed in THP-1-derived mDCs, approximately fourfold and twofold compared to THP-1-derived iDCs and THP-1 cells, respectively.

### Preparation and characterization of cyclooctyne-functionalized MNPs [35]

The MNPs used in this work are spherical iron oxide MNPs with a mean diameter of 13 nm, obtained as previously described by our group [36, 37]. The MNPs are coated with an amphiphilic polymer–poly(maleic anhydride-alt-1-octadecene), PMAO—modified with a fluorescent dye, tetramethylrhodamine 5-(6)-carboxamide (TAMRA), cadaverine to allow MNP tracking in vitro by fluorescence microscopy and flow cytometry. The maleic anhydride moieties of the polymer were hydrolyzed under alkaline conditions to yield carboxylic groups on the MNP surface for subsequent functionalization with an amino-poly(ethylene glycol) (PEG) and a cyclooctynylamine (CO) derivative, respectively (vide infra).

**Fig. 3:**
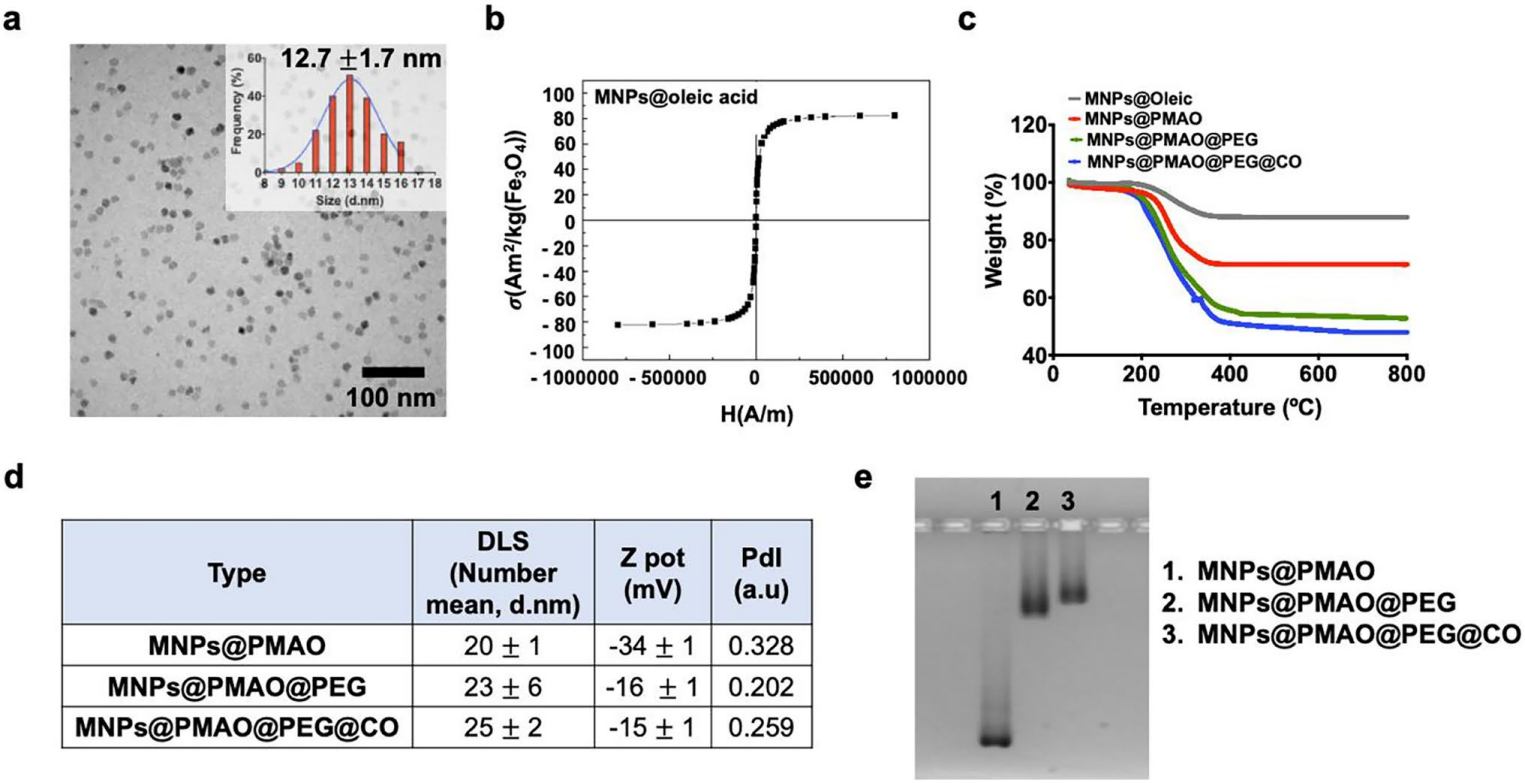
Physicochemical characterization of MNPs. **a** TEM images and **b** magnetization curve of as-synthesized MNPs (MNPs@oleic acid). **c** Thermogravimetric analysis of the different types of MNPs. **d** Dynamic light scattering (DLS) and $\zeta$-potential measurements. **e** Electrophoresis in agarose gel (1%) for 45 min at 90 V

Bioorthogonal click reaction of MNPs on living cell membranes requires the use of strained alkynes for the SPAAC reaction with azide groups. However, most of the cyclooctynes described in the literature have a pronounced hydrophobic character; in fact, the higher their hydrophobicity, the higher the reaction rate toward azides [47]. This hydrophobic character can have a negative effect on the stability of nanoparticles in biological media. For this reason, the MNPs were functionalized in a two-step process (see Fig. S1, Supplementary Information), by firstly introducing an amino-poly(ethylene glycol) (PEG) derivative to improve the colloidal stability of the MNPs, followed by the incorporation in a second step of a cyclooctynylamine derivative (CO) bearing a short ethylene glycol chain, as described in our previous work [37]. The cyclooctyne-functionalized MNPs (MNPs@PMAO@PEG@CO) had a specific absorption rate (SAR) of 112 W/g, measured at a frequency of 418 kHz and a field amplitude of 23.9 kA/m. A detailed physicochemical characterization of the MNPs is provided in Fig. 3.

The correct functionalization was confirmed qualitatively after each step by $\zeta$-potential measurements and electrophoresis in agarose gel. Moreover, from thermogravimetric analysis, we could obtain a quantitative estimation of the extent of functionalization: from the initial 3740 COOH groups present on each MNP, 1450 were modified with PEG, while in the second step approximately 760 CO moieties were introduced per MNP. For a detailed description of the calculation of the number of ligands per MNP from TGA, see page 14 in the Electronic Supplementary Information of reference [37].

### Optimization of metabolic glycoengineering and labeling of cell membrane with DBCO

The sialic acid metabolic pathway has been employed for the labeling of cell membranes, specifically for integrating azides into the glycocalyx. This process, known as metabolic glycoengineering, is initiated using a biosynthetic precursor that contains an azide functional group. For instance, in this work we used Ac_4_ManNAz, which is a synthetic molecule that can be absorbed by cells and effectively hydrolyzed to N-α-azidoacetylmannosamine (ManNAz) by cytosolic esterases. ManNAz is subsequently converted to sialic acid via five enzymatic steps and conjugated to the end of the sugar chains, displaying end azide reporter groups [35, 48]. It is noteworthy that the integration of azide reporters on cell membranes can be regulated in a dose-dependent manner, by adjusting the concentration of the azide precursor and/or the incubation time [35, 49].

In our previous work, the introduction of bioorthogonal azide reporters on the surface of different living cells (MCF7, HCT116, and A549) was optimized considering three different parameters: the concentration of the azide precursor, the incubation time, and the effect of the metabolic glycoengineering process on cell viability [35]. Based on these previous results, we selected for the subsequent experiments an incubation time of 48 h with Ac_4_ManNAz. First, azide generation on cell surfaces was initially evaluated by flow cytometry, after SPAAC reaction with a fluorescent dibenzyl cyclooctyne (DBCO-PEG_4_-5/6-Sulforhodamine B). DBCO is mostly used for fluorescent labeling and detection of azide groups via SPAAC reaction between alkyne and azide groups [35, 48, 50]. Upon incubation with the presence of Ac_4_ManNAz (10, 20, 50, and 100 µM) for 48 h, the amount of azide groups expressed on the cell membrane also increased (see Fig. S3, Supplementary Information), demonstrating a precursor dose-dependent generation of these artificial reporters, which is in line with previous literature reports [35, 51, 52].

### Labeling of cell membrane with MNPs

The MTS assay was used to evaluate the impact of MNPs and the metabolic glycoengineering process on cell viability. We do not observe cytotoxic effect upon 30 min of incubation with 100 µg_Fe_/mL, with or without the previous incubation with Ac_4_ManNAz for 48 h (Fig. S4, Supplementary Information). These results are in concordance with our previous work, since no significant cytotoxicity was observed at concentrations up to 150 µg Fe/mL using different cancer cell lines [35].

**Fig. 4:**
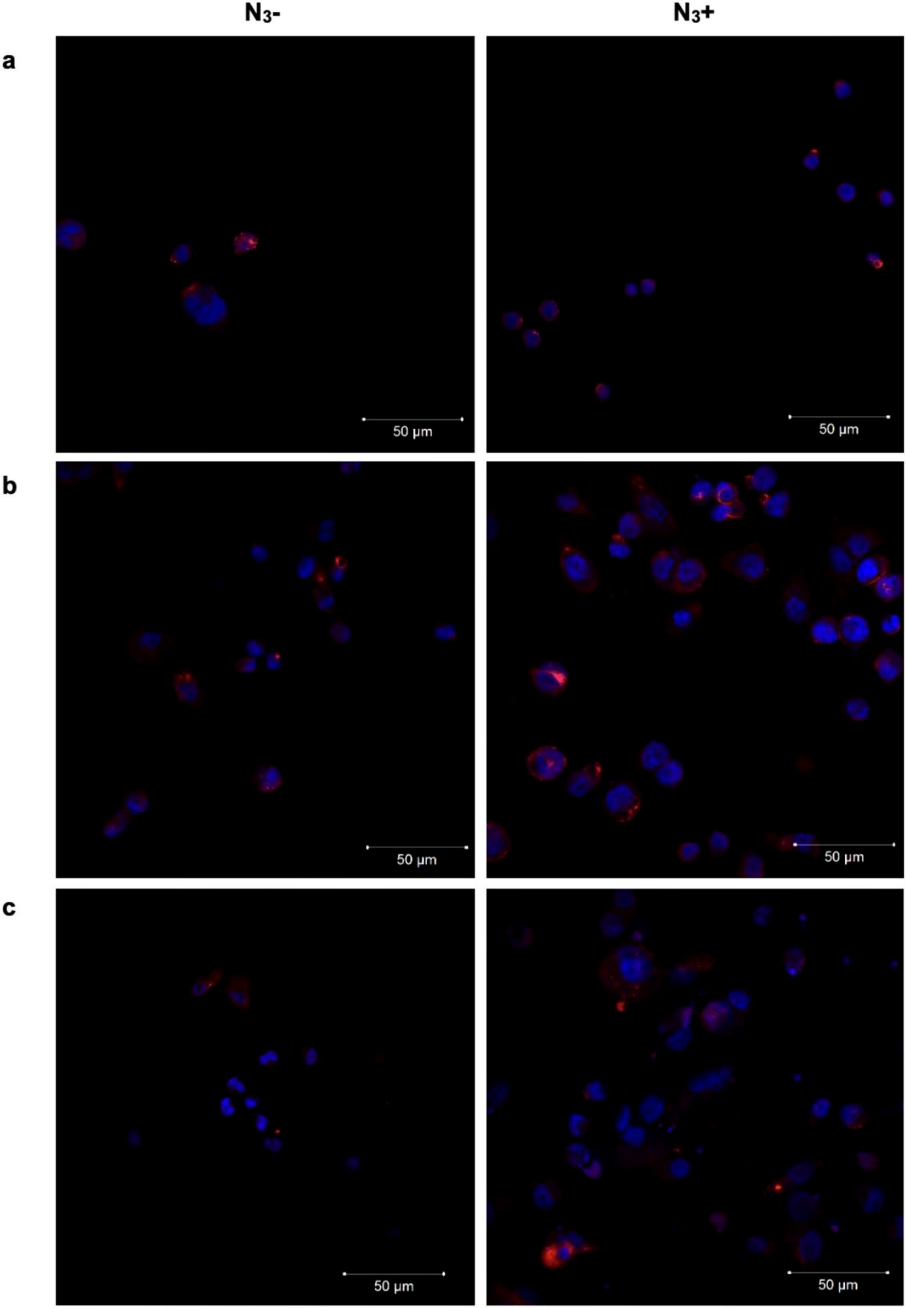
Cell membrane labeling with MNPs for 30 min in THP-1 cells and THP-derived DCs. Z-stack confocal microscopy images of the cell membrane labeling with 100 μg_Fe_/mL of MNPs in **a** THP-1 cells, **b** THP-1-derived iDCs and in **c** THP-1-derived mDCs, with the absence (N_3_-) or presence (N_3_ +) of azide groups. Red: MNPs@PMAO@PEG@CO; Blue: Hoechst 33258 (nuclei staining)

To perform the SPAAC bioorthogonal chemistry, THP-1 cells and THP-1-derived DCs expressing azide groups were incubated with the MNPs functionalized with cyclooctyne. Cells were treated with 100 μM of Ac_4_ManNAz for 48 h and then incubated for 30 min with 100 µg_Fe_/mL of MNPs, in RPMI without FBS at 37 °C. RPMI serum-free was used to circumvent any potential adverse impact that the formation of serum protein corona on the MNPs surface might have on the click reaction due to de steric hindrance between the two bioorthogonal partners [35, 53]. Control experiments using cells without Ac_4_ManNAz pre-treatment (N_3_-) were also performed. To validate the correct expression of azide groups, a positive control was included, incubating the cells with 20 μM of DBCO (DBCO-PEG_4_-5/6-TAMRA) for 30 min under the same experimental conditions. First, confocal fluorescence microscopy was used to analyze the labeling of the cell membrane with MNPs and DBCO. In the presence of previous incubation with azide groups (N_3_ +) for 48 h, as can be noted, cell labeling for 30 min with DBCO shows an increase of fluorescence signal at the cell membrane due to the covalent bonding between the alkyne and azide groups (Fig. S5, Supplementary Information). The MNPs were functionalized as described above with a fluorescent dye, TAMRA, and with a cyclooctyne to promote the click chemistry reaction. We denoted a cell membrane labeling more evident in cells treated with Ac_4_ManNAz in confocal microscopy images (Fig. 4). These results were comparable to the ones obtained in our previous work, in which prolonged MNPs incubation time seemed to enhance the click reaction but simultaneously increased non-specific interactions of the MNPs with cells lacking azide expression [35].

Consequently, to decrease non-specific interactions of the MNPs with the cell membrane, both the concentration of MNPs (from 100 to 10 µg_Fe_/mL) and the incubation time (30–10 min) were reduced. We therefore incubated THP-1 cells and THP-1-derived DCs with 10 μg _Fe/_/mL of MNPs for 10 min, which leads to a very low fluorescence signal at the cell membrane, probably due to the dispersion of the MNPs throughout the membrane surface (see Fig. S6 Supplementary Information). This result can also be attributed to the resolution limits of fluorescence microscopy, which does not allow to discriminate 13 nm diameter MNPs on fully spread cell membranes [30, 35].

**Fig. 5:**
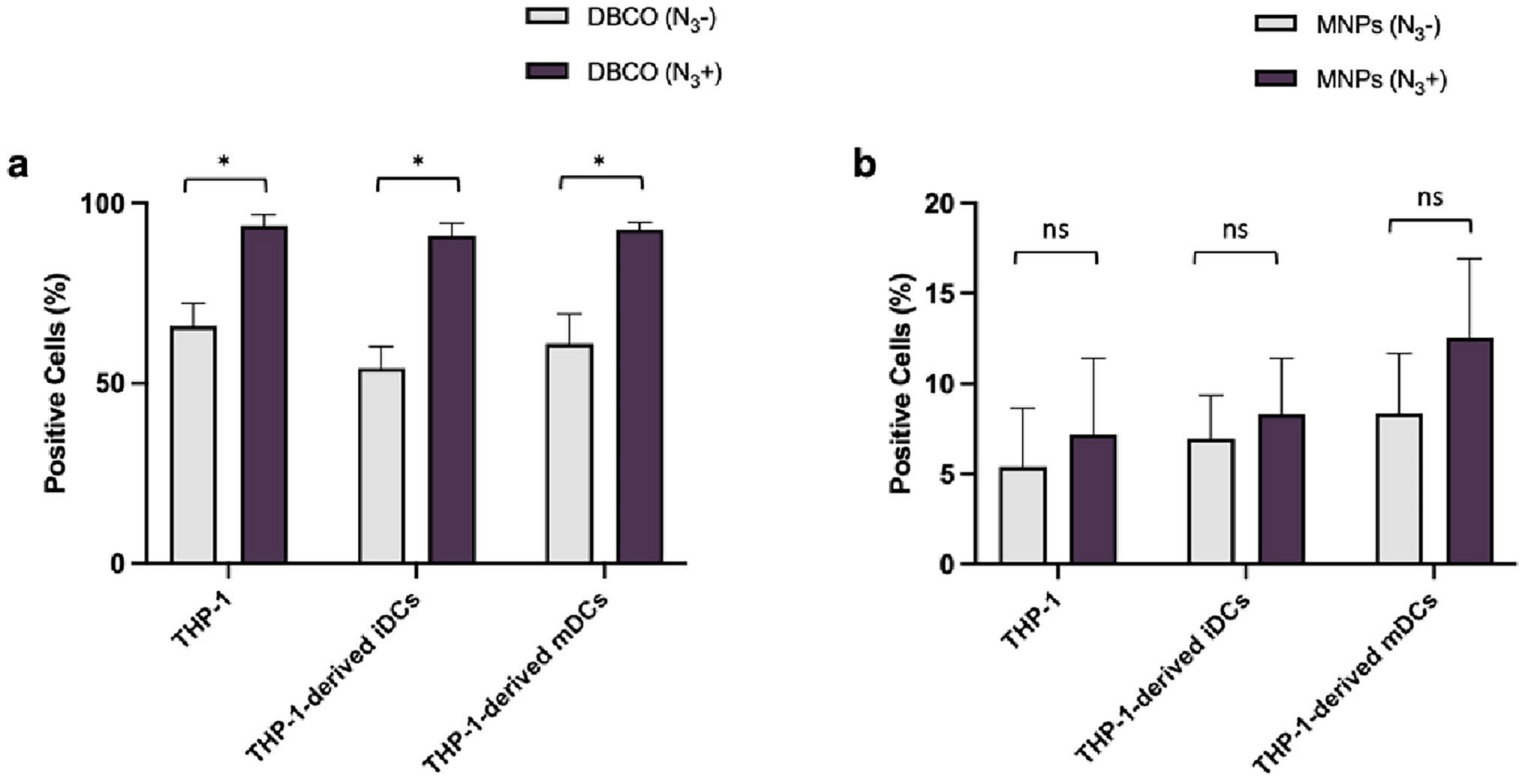
Cell membrane labeling with DBCO and with MNPs in THP-1 and THP-1-derived DCs. Cell membrane labeling with **a** DBCO-PEG_4_-5/6-TAMRA and with **b** MNPs in the absence (N_3_−) or presence (N_3_ +) of azide groups. Statistical differences were observed between cells membrane labeling with DBCO (*p < 0.05; ns—not statistically significant; two-way ANOVA—mixed-effects analysis with Tukey’s multiple comparison test). Data represent the mean value ± the standard error of the mean of at least two biologically independent experiments with two technical replicates for each

Considering these limitations, flow cytometry was used as an alternative and quantitative technique that allows to determine the total fluorescence provided by the MNPs after their interaction with cells. The bioorthogonal click reactivity of the MNPs toward azide-labeled cell membranes was evaluated by incubating THP-1 cells and THP-1-derived DCs with 10 μg_Fe/_mL of MNPs for 10 min. Cell labeling for 30 min with 20 μM of DBCO was used as a control. The results observed in Fig. 5 were compared with the interaction of MNPs and DBCO-PEG4-5/6-TAMRA with cells without the prior Ac_4_ManNAz treatment (N_3_−). The labeling of DBCO-PEG4-5/6-TAMRA on cell membrane in samples treated with Ac_4_ManNaz (N_3_+) shows statistically significant differences, confirming the click chemistry reaction between DBCO fluorescent dye and azide-labeled THP-1 cells and THP-1-derived DCs (Fig. 5a). We did not observe statistically significant differences between MNPs with (N_3_+) or without (N_3_−) the presence of azide groups, although a slight increase of cell membrane labeling in cells treated with Ac_4_ManNAz is evident. This would allow us to maximize the effect of the localized MH on the cell membrane for siRNA transfection using a minimal amount of MNPs (Fig. 5b). Concerning these results, we determine that cells with Ac_4_ManNAz treatment show an improvement of cell membrane labeling with MNPs compared to cells without the presence of azide groups, important for future magnetic hyperthermia experiments [30].

### *IDO1* gene silencing enhanced by magnetic hyperthermia

IDO1 is a rate-limiting enzyme related to tryptophan metabolism, which contributes significantly to immune suppression in the TME. The depletion of tryptophan is associated with the suppression of effector T cells activity, the differentiation of naïve T cells to Treg cells, and the recruitment of MDSCs to tumor vasculature [15, 54]. In fact, IDO1 activity is upregulated in many types of cancer and is associated with a poor prognosis [55]. Previous studies reported that the inhibition of IDO1 in DCs is considered a promising strategy to promote the efficacy of DC-based therapy [24]. Novel strategies to deliver TNAs, such as siRNA, are required to promote transfection in DCs and other primary cells of the immune system [56]. DCs are difficult to transfect, and the efficiency of transfection is low using commercially available reagents or electroporation [56–58].

In this work, we used MH to transfect siRNA against the *IDO1* gene in THP-1-derived DCs. To provide artificial azide reporters for the click immobilization of MNPs on the cell membrane, THP-1-derived DCs were incubated with Ac_4_ManNAz for 48 h before MH as described in Materials and Methods. To induce *IDO1* expression, cells were incubated with 10 ng/μL of LPS for 24 h before the MH transfection experiment. Hereafter, cells were incubated with MNPs (at 10 µg Fe/mL for 10 min) and the AMF (23.9 kA/m and 418 kHz) was applied for 30 min with pulses (each pulse with a duration of five minutes, with sixty-second pause between pulses). Transfection efficiency was compared with the positive control (Lipofectamine), under the same experimental conditions as the MH samples. Moreover, control experiments were used to verify if the presence of MNPs, with (N_3_ +) or without (N_3_-) Ac_4_ManNAz treatment and with (MH +) or without (MH-) MH application could promote the intracellular siRNA delivery (see Fig. S7 Supplementary Information). After 24 h, RNA was extracted, and RT-qPCR analysis confirmed the effectiveness of MH targeting siRNA delivery and consequently the silencing of *IDO1* gene*.*

**Fig. 6:**
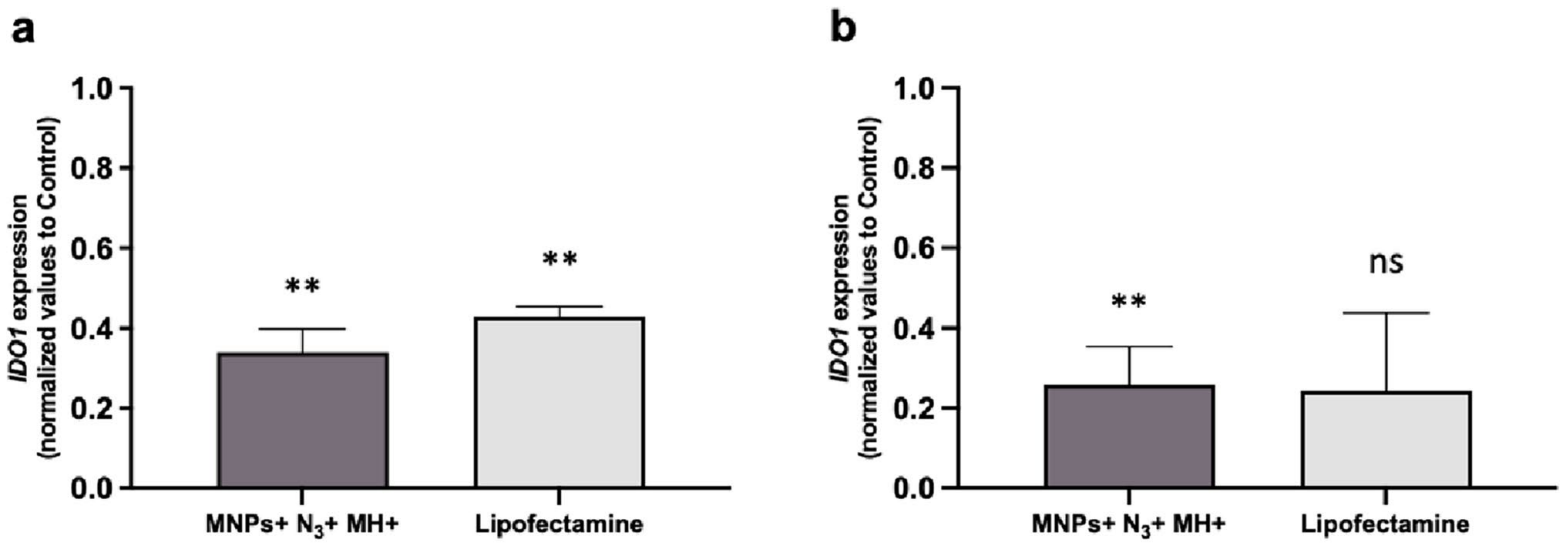
RT-qPCR assay to evaluate *IDO1* gene expression in THP-1-derived DCs. *IDO1* silencing was observed in MNPs + N_3_ + MH + and Lipofectamine samples in **a** THP-1-derived iDCs and in **b** THP-1-derived mDCs. The gene expression levels were normalized to the cells without siRNA (Control, 2^−ΔΔCt^ = 1). Black asterisks indicate statistical differences between samples and Control (**p < 0.01; ns—not statistically significant; unpaired parametric t test with Welch’s correction). Data represent the mean value ± the standard error of the mean of at least three biologically independent experiments

The results observed in Fig. 6 reveal that in cells with MNPs attached to the membrane via click chemistry and exposed to the AMF (MNPs + N_3_ + MH +), the *IDO1* expression decreased approximately 65% and 75% in THP-1-derived iDCs and THP-1-derived mDCs, respectively. This silencing effect was comparable to that attained by commercial Lipofectamine.

Our results confirmed that the MNPs immobilized on the cell membrane could act as hotspots under an AMF. The effect observed possibly reached its peak when the cell membrane glycocalyx was labeled with azides, inducing a localized heating and triggering an increase in the permeability and fluidity of the membrane, which enhances the transfection of siRNA against *IDO1* gene. However, it remains essential to tailor the dosage of silencing moieties for optimal gene silencing in specific contexts [30].

The contribution of IDO1 in immunomodulation in cancer has been explored since this enzyme depletes tryptophan in TME, which suppresses T cell proliferation and promotes the differentiation of Treg cells [14]. *IDO1* silencing can reduce this immunosuppressive effect, potentially leading to a more robust immune activation [18, 59]. For example, Endo et al*.* reported *IDO1* gene silencing in DCs derived from mouse using low doses of siRNA encapsulated in lipid nanoparticles, and the silencing efficiency was compared to commercial reagents available for transfection, such as Lipofectamine [24]. In other study, the role of *IDO1* silencing in the inhibition of lung cancer growth was demonstrated using an in vitro Lewis lung carcinoma (LLC) cell line. Moreover, T cells exhaustion during lung cancer progression was confirmed in an in vivo model using shRNA against *IDO1* gene [60]. Interestingly, Sioud et al*.* produced a safe IDO-silenced DC vaccine to activate allogeneic T cells in four patients with gynecological cancer to induce anticancer immunity [61]. For instance, *IDO1* silencing could enhance the immunogenic function of DCs in vitro and in vivo, and the silence effect did not affect the expression of the costimulatory molecules CD80 and CD86 [61, 62]. Zheng et al*.* observed the reduction of the tumor size and the decrease of CD4 + and CD8 + apoptosis in a mouse model of breast cancer using DCs loaded with tumor antigens and siRNA targeting *IDO1* gene [63]. These findings suggest that silencing *IDO1* could potentially enhance the effectiveness of human DC vaccines [19]. Overall, those studies described the *IDO1* gene as a potential target for gene therapy in cancer.

### Effect of IDO1 gene silencing on the gene expression of pro-inflammatory and anti-inflammatory cytokines

The expression of cytokines, such as IL-6 [64], TNF-α [65], IL-12A [66], and IL-10 [67], is crucial for the role of DCs in the immune response, since those cytokines are able to modulate the balance between pro-inflammatory and anti-inflammatory status. IL-6 plays a critical role in immune responses, inflammation, and hematopoiesis [68]. The expression of this cytokine is upregulated in DCs upon activation by various stimuli (e.g., pathogen-associated molecular patterns recognized by Toll-like receptors) and is involved in the differentiation of naïve T cells into Th17 cells [64, 69]. TNF-α is involved in the activation of macrophages and T cells, promotes inflammation, and enhances the maturation and antigen-presenting capabilities of DCs [70, 71]. This cytokine is produced by activated DCs, and its expression is induced in response to contact with T cells, cytokines, or pathogens [72]. IL-12A is generally produced by activated DCs and/or mDCs and is essential to promote the differentiation of Th1 cells and the cytotoxic functions of NK cells and cytotoxic T cells [73, 74]. The expression of IL-12A in DCs is upregulated in response to exogenous stimuli (e.g., pathogen products) and interactions with activated T cells [75, 76]. Contrary to those cytokines, IL-10 regulates immune responses by suppressing the expression of pro-inflammatory cytokines (e.g., IL-6 and TNF-α) and can modulate the function of DCs while inhibiting their maturation and reducing their capacity to present antigens [67, 77]. IL-10 expression can be induced in response to immune regulatory signals or pathogens and is important to prevent excessive inflammatory responses [78, 79]

**Fig. 7:**
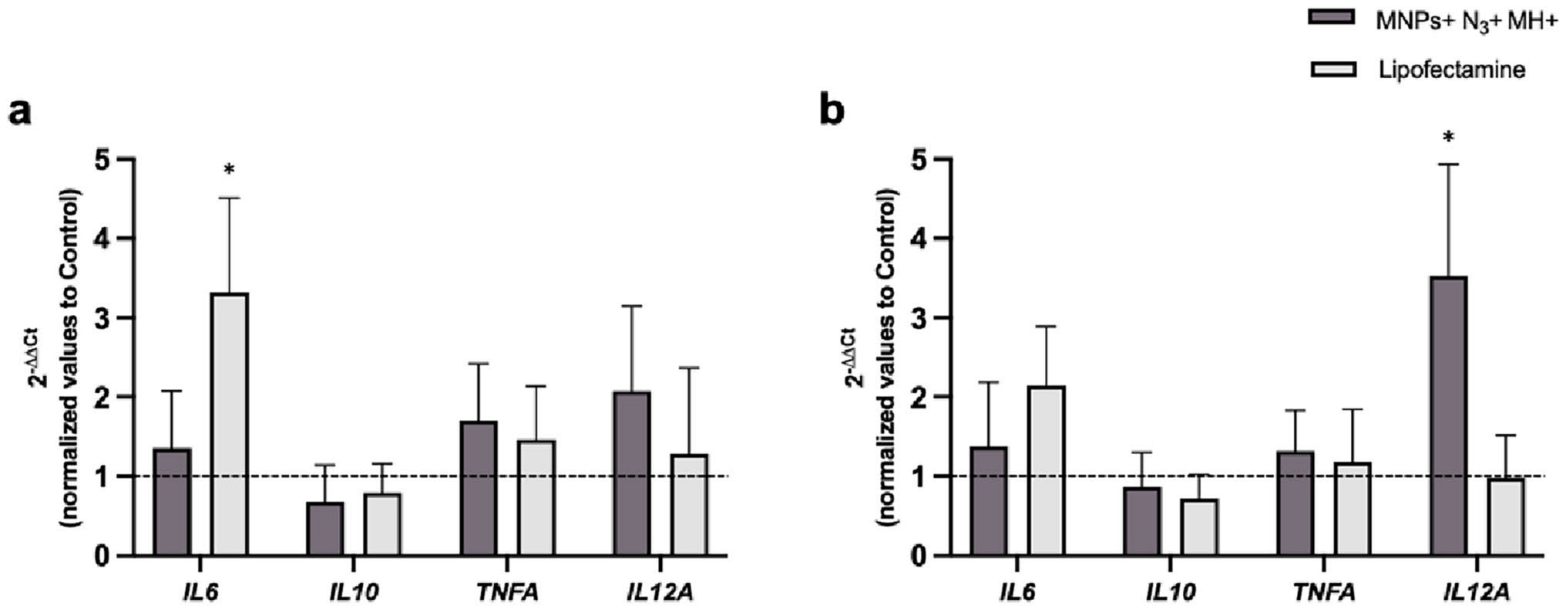
RT-qPCR analysis of gene expression of *IL-6*, *IL-10*, *TNFA,* and *IL-12A* after *IDO1* silencing. The gene expression levels were normalized to cells without siRNA (Control, 2^−ΔΔCt^ = 1). Black asterisks indicate statistical differences between Lipofectamine sample and Control in **a** THP-1-derived iDCs; and between MNPs + N_3_ + MH + and Control in **b** THP-1-derived mDCs (*p < 0.05; two-way ANOVA—Mixed-effects analysis with Tukey’s multiple comparison test). Data represent the mean value ± the standard error of the mean of at least three biologically independent experiments with two technical replicates for each. Overall, *IDO1* gene silencing promotes *IL-6*, *TNFA,* and *IL-12A* upregulation and *IL-10* downregulation in MNPs + N_3_ + MH + and Lipofectamine samples

Considering all this, we evaluated the effect of *IDO1* silencing on the expression of genes encoding pro-inflammatory cytokines IL-6, IL-12A, and TNF-α, and anti-inflammatory cytokine IL-10 in all conditions previously tested (see Fig. S8, Supplementary Information). As observed in Fig. 7, an upregulation of mRNA levels of *IL-6, TNFA,* and *IL-12A* and a downregulation of *IL-10* expression are observed in THP-1-derived DCs transfected with MNPs + N_3_ + MH + or Lipofectamine, which are consistent with previous reports (see below) regarding *IDO1* gene silencing.

Silencing the *IDO1* gene in DCs could significantly impact their function and the expression of these cytokines [18, 61, 80]. For instance, researchers have been exploring the inhibition of IDO1 activity and its impact on the cytokine’s expression. A previous study conducted in LLC-bearing mice treated with shRNA against the *IDO1* gene shows an increase in the production of pro-inflammatory cytokines TNF-α and IL-2 compared to controls, which suggests that this treatment may recover cytokine secretion in tumors in vivo [60]. Heidari et al*.* described the effect of IDO1 suppression on the immunomodulatory function of adipose-derived mesenchymal stem cells in cancer, which leads to a downregulation of the expression of the immunoregulatory mediators studied, namely Gal‐3, HGF, TGF‐β, and IL-10 [81]. Several studies reported that the induction of IL-10 expression in monocytes promotes Treg cells to express IL-10 and TGF‐β and consequently suppresses the activity of DCs and Th1 cells [82, 83]. Ravishankar et al*.* denoted a significant reduction in IL-10 and TGF‐β protein relative levels and an increase in mRNA relative levels of pro-inflammatory cytokines TNF-α, IL-6, and IL-12 in mice [84]. The literature has been recognized that DCs‑secreted IL-10 promotes the development of Treg cells, while IL-12 induces the differentiation of CD4 + T cells into Th1 cells [85, 86]. Thus, IDO1 activity suppresses effector T cell responses, which promotes the differentiation and activation of Treg cells and inhibits the production of IL-6 by DCs [87]. Recently, the impact of IDO1 on the production of secreted cytokines was studied using stable DCs lines with both gain and reduction of IDO1 function, established by recombinant DNA technique. The reduction of IDO1 function improves the secretion of Th1 cell polarizing IL-12 and decreases Treg cell polarizing IL-10 from DCs, which is important to understand the tolerogenic regulation of DCs‑mediated T cells differentiation by IDO1 [80]. These data confirm that tryptophan depletion facilitates the production of anti-inflammatory cytokines by IDO-expressing DCs, which promotes the recruitment of Treg cells, and suppresses T cell activation and proliferation [61, 80]. Additionally, it has been widely reported that DCs with elevated kynurenine levels (tolerogenic stage) drive T cells differentiation to Treg cells and induce apoptosis in effector T cells. In contrast, DCs with low IDO expression (immunogenic stage) hamper Treg cells development and reduce T cells apoptosis [80, 88].

### Cells’ viability assessment post-transfection of siRNA

**Fig. 8:**
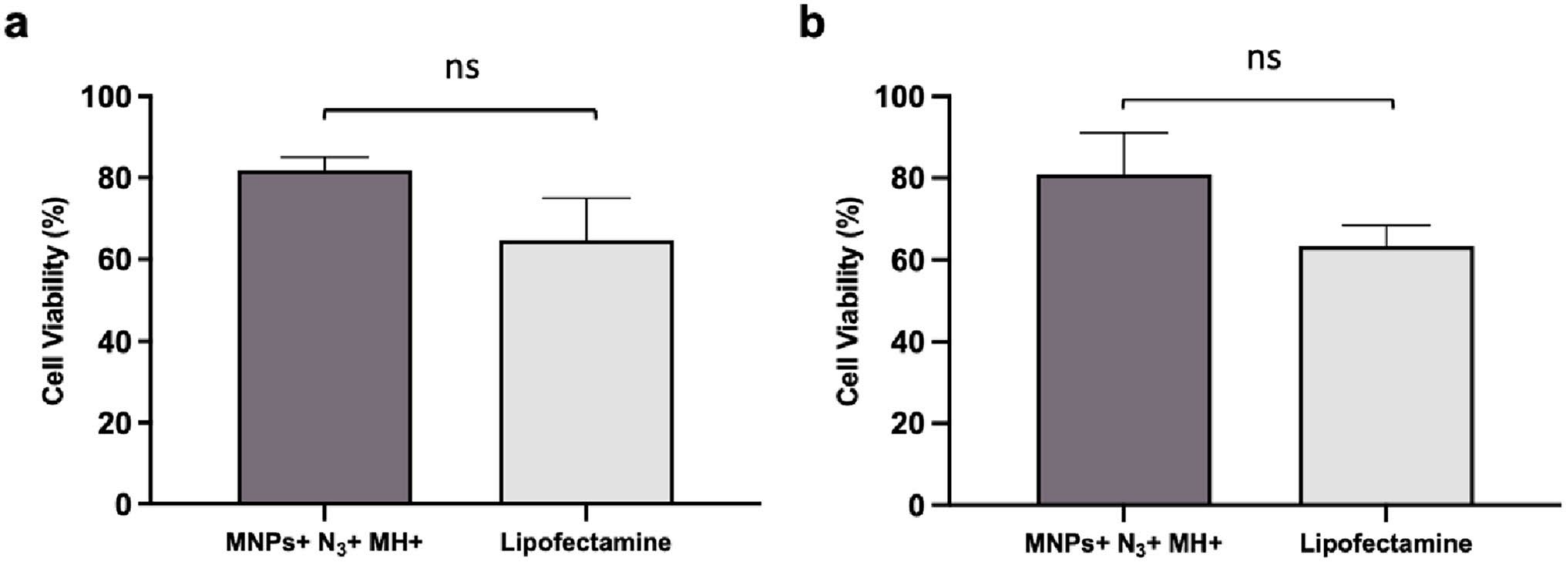
Cell viability analysis post-MH experiment via MTS assay in THP-1-derived DCs. The conditions tested were normalized to cells without siRNA (Control). Statistical differences were not observed between MNPs + N_3_ + MH + and Lipofectamine in **a** THP-1-derived iDCs and in **b** THP-1-derived mDCs (ns—not statistically significant; unpaired parametric t test with Welch’s correction). Data represent the mean value ± the standard error of the mean of at least three biologically independent experiments with two technical replicates for each

Many of the commercially available transfection methods are known for their high impact on cell viability. Several studies have described the cytotoxicity of cationic lipid reagents and the impact of electroporation on cell survival [27, 89]. Therefore, the impact on cell viability was assessed in THP-1-derived DCs upon the transfection of siRNA against the *IDO1* gene in all conditions tested (Fig. S9, Supplementary Information). Figure 8 reveals that Lipofectamine induces a statistically significant reduction of cell viability of approximately 40% in THP-1-derived DCs, not observed for MNPs + N_3_ + MH + (cell viability of 80%).

As observed in Fig. 8, when compared to our MH strategy, Lipofectamine has a more cytotoxic effect in THP-1-derived DCs. Indeed, using our MH transfection strategy, a lower impact on cell viability was observed (81% of cell viability) (Fig. 8), which might be related to the *IDO1* silencing effect. IDO1 is an enzyme involved in immune regulation and modulation of metabolic pathways, such as the catabolism of tryptophan through the kynurenine pathway [12]. Silencing of the *IDO1* gene results in higher levels of tryptophan and reduces the production of kynurenine and its downstream metabolites, leading to shifts in cellular respiration and energy production that potentially can affect cell proliferation. For instance, the kynurenine pathway contributes to NAD + synthesis and reduced activity of this pathway, which can impact NAD + levels, influencing metabolic processes like glycolysis, oxidative phosphorylation, and DNA repair mechanisms [90, 91]. In some contexts, silencing of the *IDO1* gene can promote cell death through various mechanisms, such as increasing pro-apoptotic factors, enhancing the activity of effector T cells and NK cells in the TME, and altering signaling pathways that regulate cell survival [12, 14, 59]. Taken together, these findings suggest that this novel transfection approach can attain comparable transfection compared to the commercially available reagent Lipofectamine, with less cytotoxic effect. Nonetheless, it is important to consider that part of the observed viability reduction may stem from the biological effects of the *IDO1* gene silencing itself. Further research on metabolic assessments will be important to clarify the potential contribution of IDO1 inhibition in cell proliferation and survival.

### Conclusions

IDO1 plays a critical role in modulating tryptophan metabolism, immune responses, and cell survival pathways. Silencing the *IDO1* gene impacts these processes, leading to altered metabolic states and increased cell death, particularly in contexts where *IDO1* is upregulated, such as in many cancers (e.g., colorectal carcinoma, melanoma), due to its contribution to the immunosuppressive TME.

Our results indicated an effective gene silencing in THP-1-derived DCs using MH-mediated transfection of siRNA against the *IDO1* gene via SPAAC reaction of cyclooctyne-MNPs with azide groups expressed on the cell membrane via metabolic glycoengineering. Our approach has the same silencing efficiency as Lipofectamine and shows a lower impact on cell viability due to the silencing of the *IDO1* gene when compared to this commercially available lipid cationic reagent. The MNPs act as cell membrane-localized heating sources due to their immobilization at the time of AMF application, increasing membrane fluidity and permeability, which in turn promotes the delivery of silencing moieties. Moreover, we proved that *IDO1* silencing in THP-1-derived DCs possibly leads to a shift toward a pro-inflammatory state (more immunogenic) characterized by the increase of gene expression of *IL-6*, *TNFA*, and *IL-12* and the decrease of *IL-10* mRNA levels.

IDO1 inhibition has been explored as a therapeutic strategy in cancer since it may enhance antitumor immunity by boosting the activity of DCs and other immune cells, and shifting to a pro-inflammatory state could potentiate immune responses. The reduction of the immunosuppressive effects of IDO1 can lead to enhanced activation and proliferation of effector T cells, and the specific outcomes will depend on the context in which *IDO1* is silenced (e.g., TME). Exploring our MH strategy as a universal platform for intracellular delivery in other cells that play a crucial role in TME (e.g., cancer-associated fibroblasts and tumor-associated macrophages) and adapting for more advanced cancer cell models (e.g., 3D spheroids, patient-derived organoids) are highly relevant for future applications. Indeed, metabolic glycoengineering combined with SPAAC reaction can be applied to virtually any cell line, so we expect that our approach can be extended to other types of hard to transfect cells. Moreover, combining the *IDO1* gene silencing with immune checkpoint inhibitors (e.g., anti-PD-1/PD-L1) would be useful to enhance the antitumor immune response. Overall, our findings highlight the potential of IDO1 as a therapeutic target and the employment of MH-mediated transfection for TNAs, or other molecules for targeted therapy in cancer.

## Supplementary Information

Below is the link to the electronic supplementary material.

Supplementary file1 (DOCX 2729 KB)

## Data Availability

All data generated or analyzed during this study are included in this published article [and its supplementary material files]. No datasets were generated or analyzed during the current study.
