# Supplementary material for: Heat up, silence on: IDO1 gene silencing in THP-1-derived dendritic cells triggered by magnetic hyperthermia
Source: Cancer Immunol Immunother. 2025 Aug 23;74(9):292. doi: 10.1007/s00262-025-04148-3 (PMC12374930; doi:10.1007/s00262-025-04148-3)
Supplement: Supplementary file 1 — Supplementary file1 (DOCX 2729 KB) [file 262_2025_4148_MOESM1_ESM.docx]

**Cancer Immunology, Immunotherapy (submitted in 2025) – Daniela Ferreira *et al.***

**Electronic Supplementary Material**

**Supplementary Table 1**

**Table. S1** Primers sequences used in RT-qPCR. Fwr: Forward primer. Rev: Reverse primer.

| Gene (GenBank) | Sequence (5'-3') | Product length (bp) |
| --- | --- | --- |
| *IDO1*  (NM_002164.6) | Fwr: ACTGTGTCTTGGCAAACTGGAAG  Rev: CAGCTGCTATTTCCACCAATAGAG | 141 |
| *IL6*  (NM_000600.5) | Fwr: GGT ACA TCC TCG ACG GCA TCT  Rev: TCT TTG CTG CTT TCA CAC AT | 76 |
| *IL10*  (NM_000572.3) | Fwr: GGT TGC CAA GCC TTG TCT GA  Rev: CCC CCA GGG AGT TCA CAT G | 106 |
| *TNFA*  (NM_000594.4) | Fwr: CCA GGC AGT CAG ATC ATC TTC TC  Rev: TAT CTC TCA GCT CCA CGC CA | 143 |
| *IL12A*  (NM_001397992.1) | Fwr: GAATTTTACCCTTGCACTTCTGA  Rev: CAGGCAACTCCCATTAGTTATGA | 156 |
| *18S*  (NR_003286.4) | Fwr: GTAACCCGTTGAACCCCATT  Rev: CCATCCAATCGGTAGTAGCG | 151 |

siRNA target sequence against *IDO1* gene: 5’ AGAAAGAGUUGAGAAGUUA 3’

**Supplementary Figure 1**

**Fig. S1** Functionalization of MNPs with amino-poly(ethylene glycol) (PEG) and cyclooctynylamine (CO) derivatives. Scheme of MNPs functionalization with amino-poly(ethylene glycol) (PEG) and cyclooctynylamine (CO) derivatives in a two-step process.

**Supplementary Figure 2**


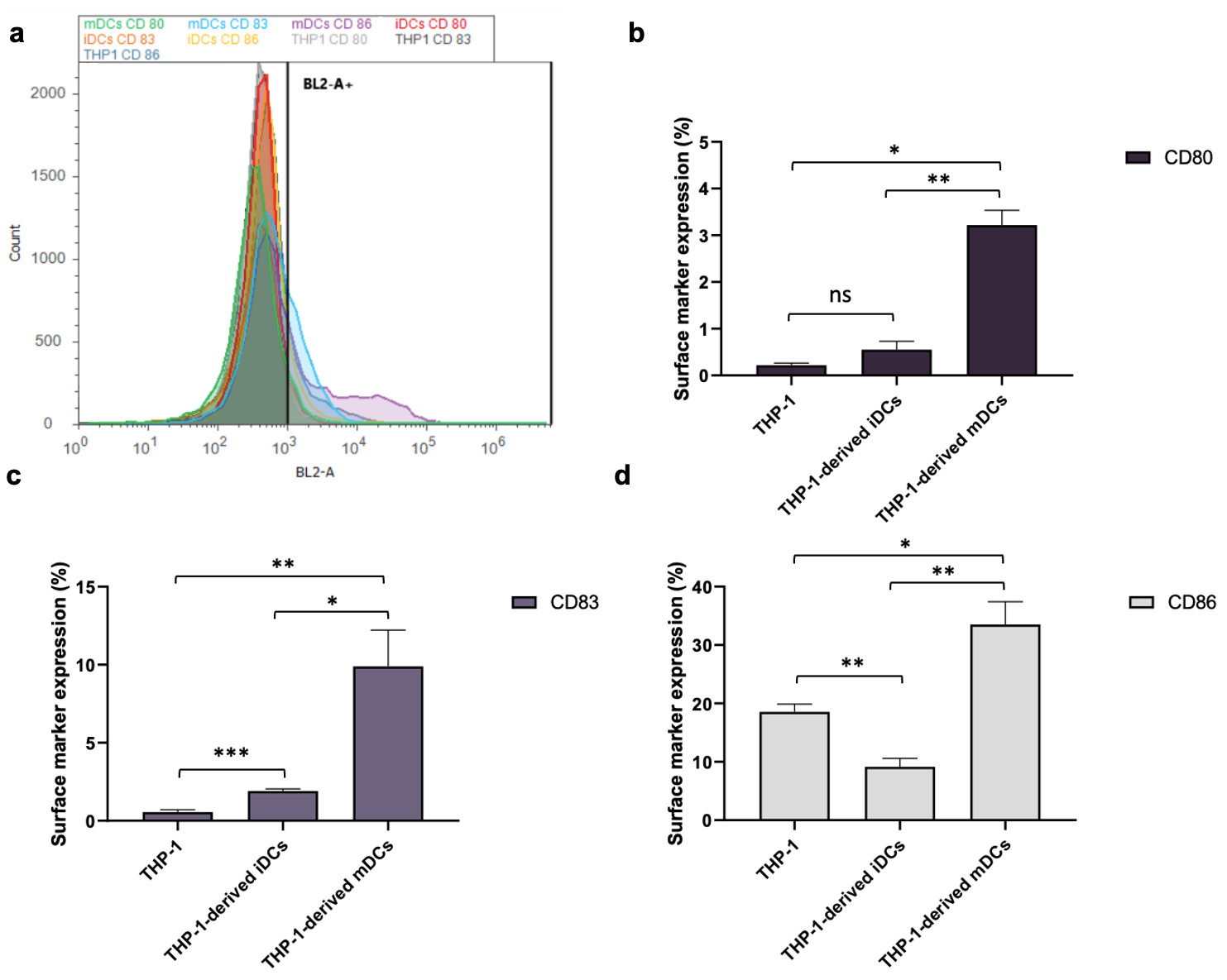


**Fig. S2** Characterization of the expression of cell membrane receptors in THP-1 cells and in THP-1-derived DCs. **a)** Histogram of the expression of surface markers analyzed by flow cytometry in the different stages of THP-1 cells; **b)** CD80 surface marker expression; **c)** CD83 surface marker expression; **d)** CD86 surface marker expression. Statistical differences were observed between THP-1 and THP-1-derived DCs for the membrane receptors analyzed. (*p < 0.05; **p < 0.01; ***p < 0.001; ns – not statistically significant; two-way ANOVA - Mixed-effects analysis with Tukey’s multiple comparison test). Data represent the mean value ± the standard error of the mean of at least three biologically independent experiments, with two technical replicates for each.

**Supplementary Figure 3**


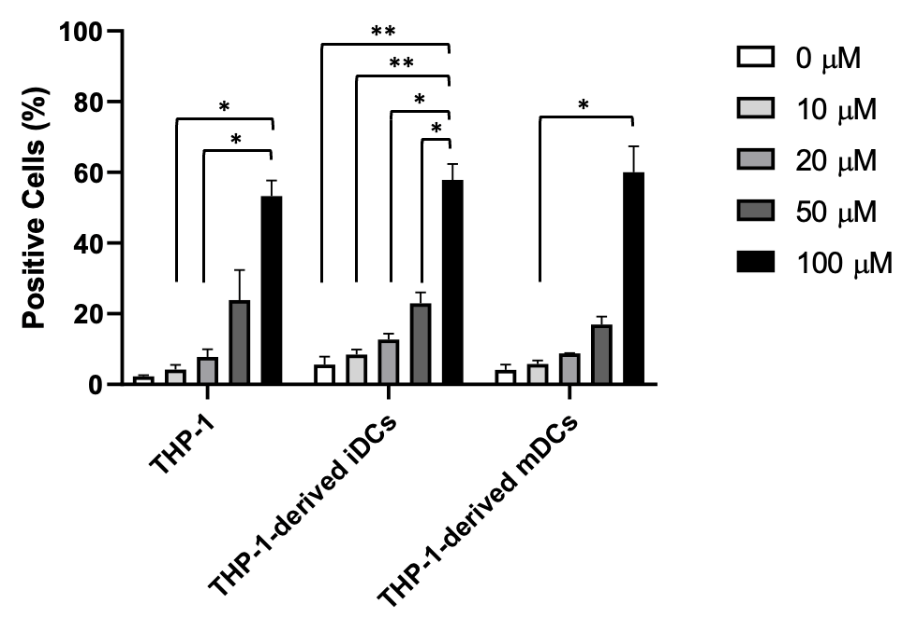


**Fig. S3** Optimization of cell membrane labeling with azide groups in THP-1 cells and THP-1-derived DCs. Different concentrations of Ac_4_ManNAz (0 to 100 $\mu$M) were used to optimize cell membrane labeling, and the expression of azide groups was evaluated using flow cytometry after SPAAC reaction with DBCO-PEG_4_-5/6-Sulforhodamine B (20 $\mu$M). Statistical differences were observed between cell membrane labeling with DBCO (*p < 0.05; *p < 0.01; ns – not statistically significant; two-way ANOVA - Mixed-effects analysis with Tukey’s multiple comparison test). Data represent the mean value ± the standard error of the mean of two biologically independent experiments with two technical replicates for each.

**Supplementary Figure 4**

**Fig. S4** Cell viability analysis via MTS assay in THP-1 cells and THP-1-derived DCs. The conditions tested were normalized to untreated cells. N_3_+: cells treated with 100 $\mu$M of Ac_4_ManNAz for 48 h; MNPs+ N_3_-: cells incubated for 30 minutes with 100 $\mu$g_Fe_/mL of MNPs; MNPs+ N_3_+: cells treated with 100 $\mu$M of Ac_4_ManNAz for 48 h and incubated for 30 minutes with 100 $\mu$g_Fe_/mL of MNPs. Statistical differences were not observed between the samples and untreated cells. Data represent the mean value ± the standard error of the mean of two biologically independent experiments with two technical replicates for each.

**Supplementary Figure 5**


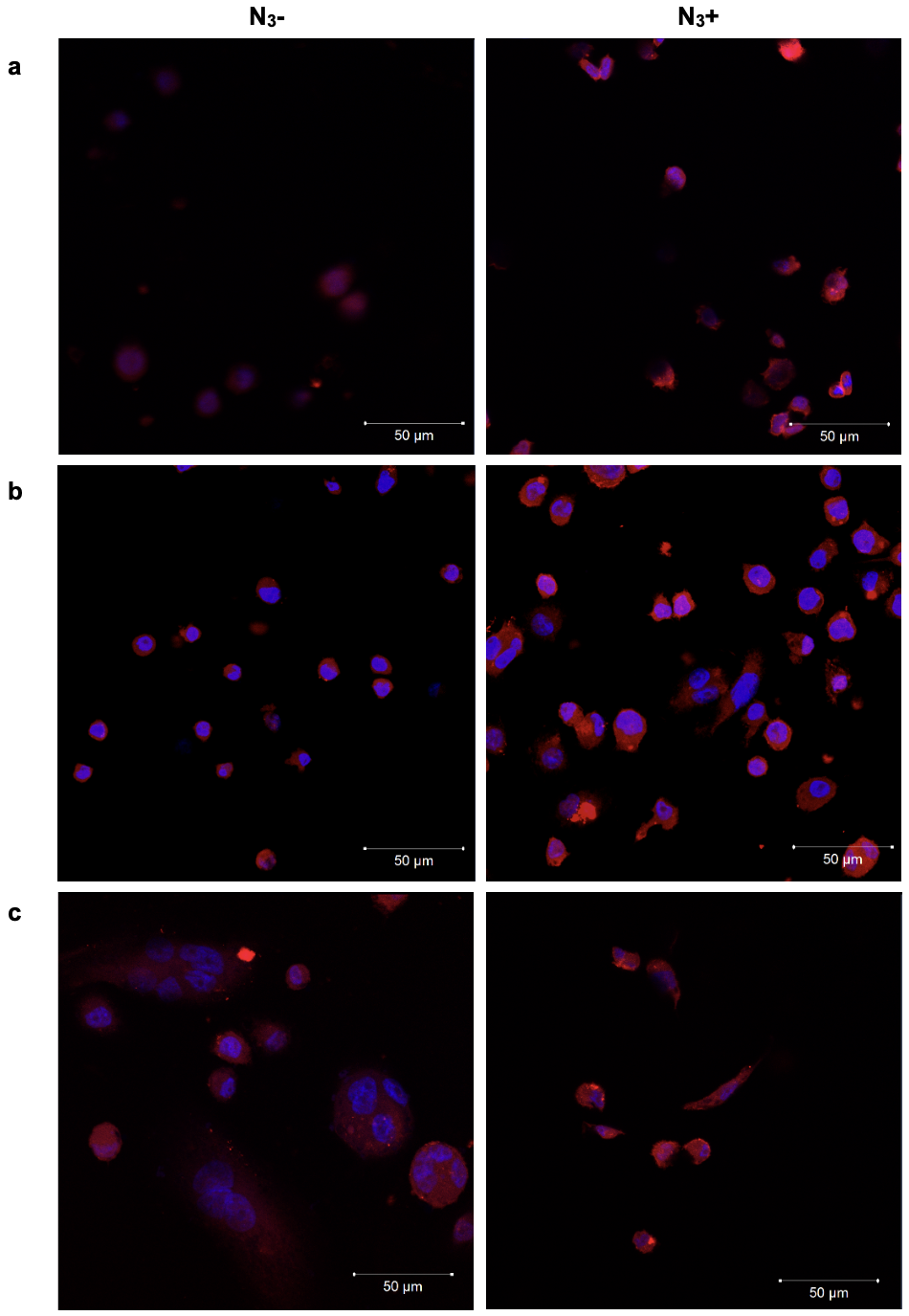
**Fig. S5** Cell membrane labeling with DBCO for 30 minutes in THP-1 cells and THP-derived DCs. Z-stack confocal microscopy images of the cell membrane labeling with 20 $\mu$M of DBCO-PEG_4_-5/6-TAMRA in **a)** THP-1 cells, **b)** THP-1-derived iDCs and in **c)** THP-1-derived DCs, with the absence (N_3_-) or presence (N_3_+) of azide groups. Red: DBCO-PEG_4_-5/6-TAMRA; Blue: Hoechst 33258 (nuclei staining)

**Supplementary Figure 6**


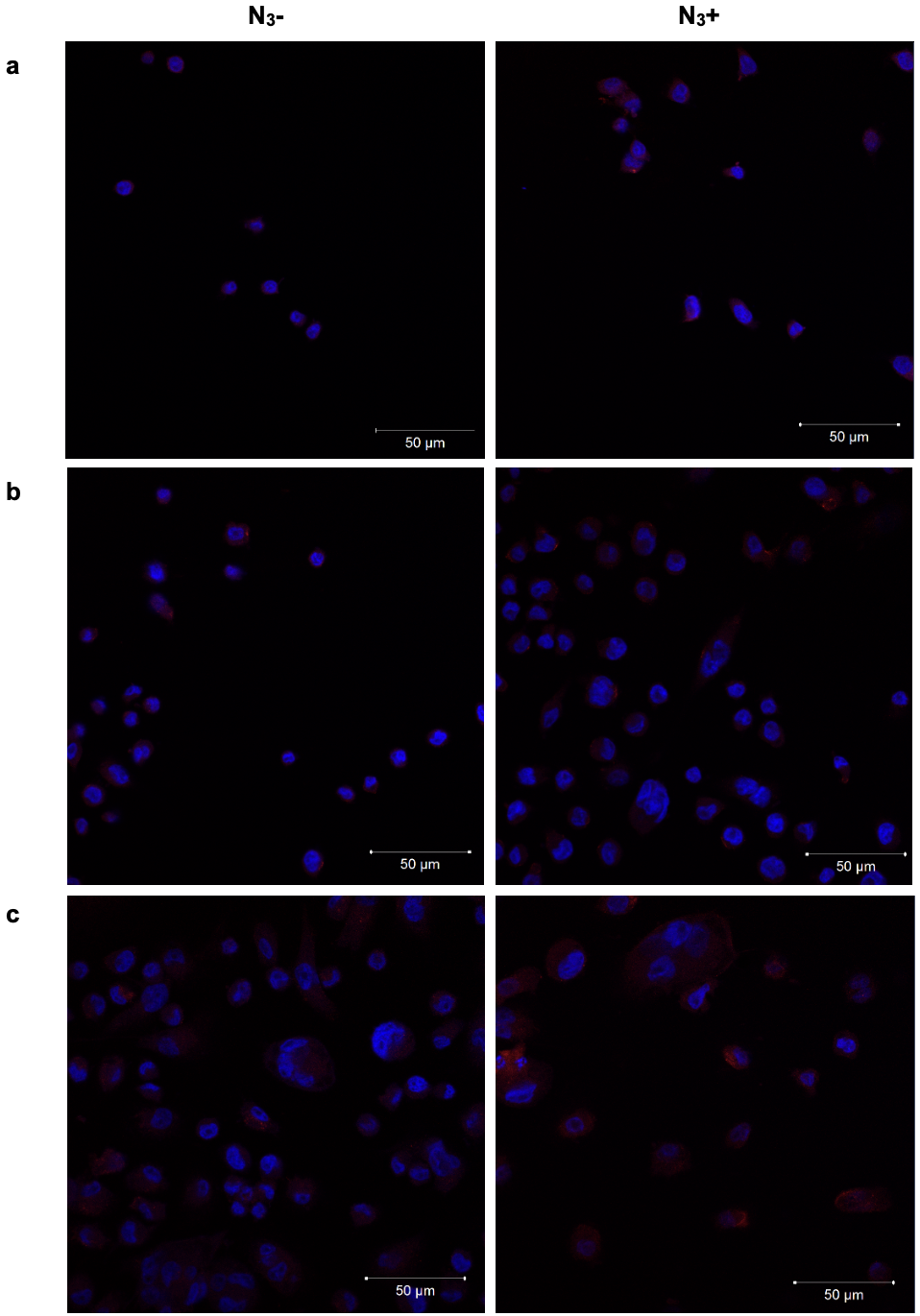
**Fig. S6** Cell membrane labeling with MNPs for 10 minutes in THP-1 cells and THP-derived DCs. Z-stack confocal microscopy images of the cell membrane labeling with 10 $\mu$g_Fe_/mL of MNPs in **a)** THP-1 cells, **b)** THP-1-derived iDCs, and in **c)** THP-1-derived DCs, in the absence (N_3_-) or presence (N_3_+) of azide groups. Red: MNPs@PMAO@PEG@CO; Blue: Hoechst 33258 (nuclei staining)

**Supplementary Figure 7**


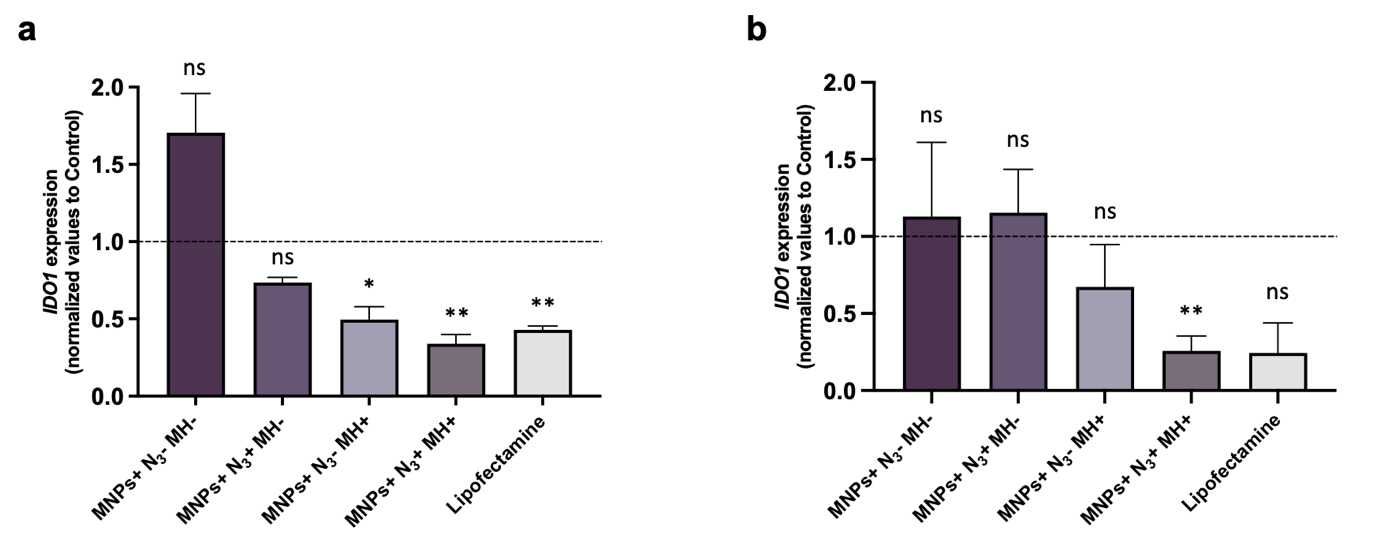
**Fig. S7** RT-qPCR assay to evaluate *IDO1* gene expression in the different conditions tested. *IDO1* silencing was observed in all samples tested except MNPs+ N_3_- MH- in **a)** THP-1-derived iDCs; except MNPs+ N_3_- MH- and MNPs+ N_3_+ MH- in **b)** THP-1-derived mDCs. The gene expression levels were normalized to the cells without siRNA (Control, $2^{-\Delta\Delta}$^Ct^=1). Black asterisks indicate statistical differences between samples and Control (*p < 0.05; **p < 0.01; ns – not statistically significant; Unpaired parametric t-test with Welch’s correction). Data represent the mean value ± the standard error of the mean of at least three biologically independent experiments. MNPs: magnetic nanoparticles; N_3_-: absence of azide groups; N_3_+: presence of azide groups; MH-: without magnetic hyperthermia application; MH+: with magnetic hyperthermia application.

**Supplementary Figure 8**


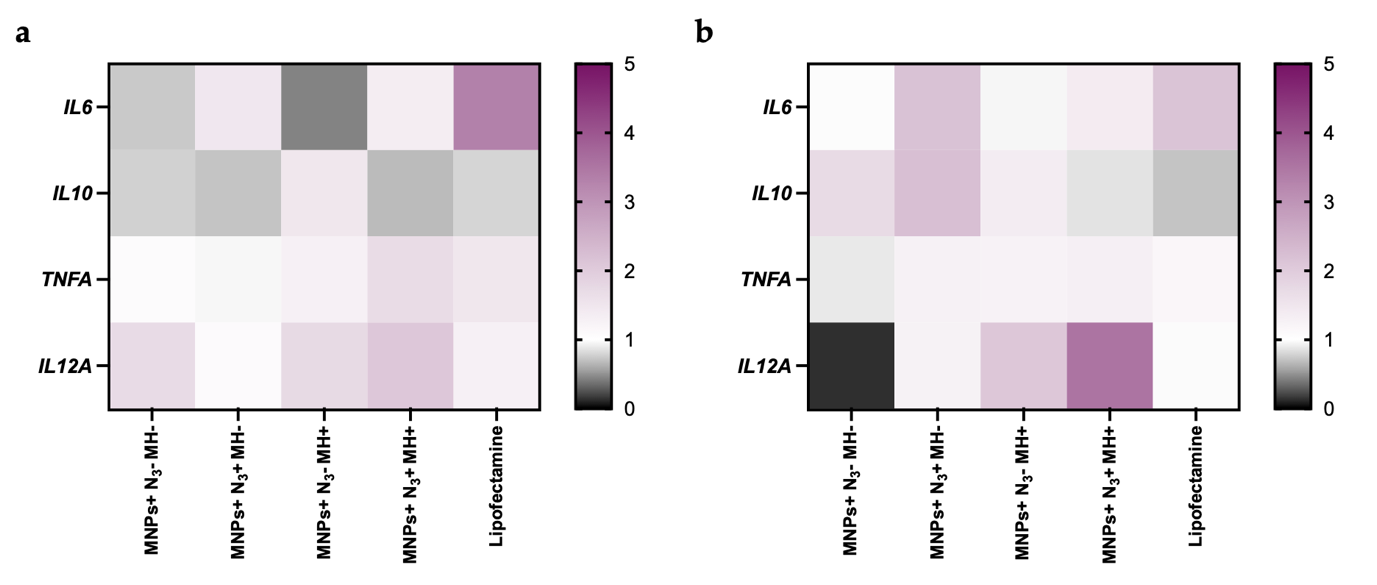
**Fig. S8** Evaluation of cytokines *IL6*, *IL10*, *TNFA,* and *IL12A* gene expression by RT-qPCR assay in different conditions tested after *IDO1* silencing. Heatmap of **a)** THP-1-derived iDCs and **b)** THP-1-derived mDCs. The gene expression of these cytokines was normalized to the cells without siRNA (Control, $2^{-\Delta\Delta}$^Ct^=1: white color); purple represents the upregulation of gene expression, and black represents the downregulation of gene expression. Data represent the mean value of at least three biologically independent experiments with two technical replicates for each. MNPs: magnetic nanoparticles; N_3_-: absence of azide groups; N_3_+: presence of azide groups; MH-: without magnetic hyperthermia application; MH+: with magnetic hyperthermia application.

**Supplementary Figure 9**


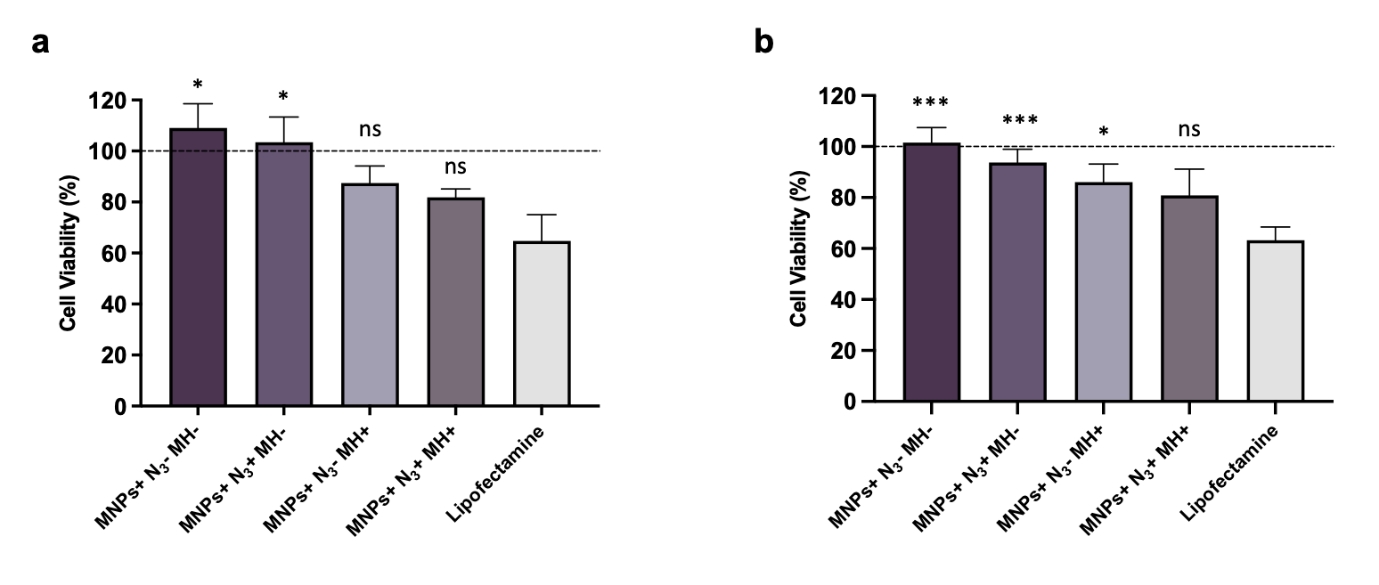


**Fig. S9** Cell viability analysis of the different samples tested post-MH experiment via MTS assay. The five conditions tested were normalized to cells without siRNA (Control). Statistical differences were observed between Control, MNPs+ N_3_- MH- and MNPs+ N_3_+ MH- samples with Lipofectamine in **a)** THP-1-derived iDCs; and between Control, MNPs+ N_3_- MH-, MNPs+ N_3_+ MH- and MNPs+ N_3_- MH- samples with Lipofectamine in **b)** THP-1-derived mDCs (*p < 0.05; ***p < 0.001; ns – not statistically significant; Unpaired parametric t-test with Welch’s correction). Data represents the mean value ± the standard error of the mean of at least three biologically independent experiments with two technical replicates for each. MNPs: magnetic nanoparticles; N_3_-: absence of azide groups; N_3_+: presence of azide groups; MH-: without magnetic hyperthermia application; MH+: with magnetic hyperthermia application.
